# The Plant Protease Inhibitor EcTI Suppresses Melanoma Progression In Vivo

**DOI:** 10.3390/molecules31162829

**Published:** 2026-08-13

**Authors:** Camila Ramalho Bonturi, Bruno Ramos Salu, Kathleen Chwen Ming Lie, Márcia Bonini, Rita de Cassia Sinigaglia, Miryam Paola Alvarez-Flores, Ana Marisa Chudzinski-Tavassi, Heloisa Sobreiro Selistre-de-Araujo, Maria Luiza Vilela Oliva

**Affiliations:** 1 Department of Biochemistry, Universidade Federal de São Paulo (UNIFESP), São Paulo 04044-020, SP, Brazil; 2 Electron Microscopy Center, Universidade Federal de São Paulo (UNIFESP), São Paulo 04044-020, SP, Brazil; 3 Centre of Excellence in New Target Discovery (CENTD), Instituto Butantan, São Paulo 05503-900, SP, Brazil; 4 Department of Physiological Sciences, Universidade Federal de São Carlos, Rodovia Washington Luis, Km 235, São Carlos 13565-905, SP, Brazil

**Keywords:** focal adhesion kinase, integrins, in vivo, in vitro, melanoma, mitochondrial dysfunction, protease inhibitor, regulated cell death

## Abstract

Melanoma dissemination depends on tumor cell plasticity, extracellular matrix remodeling, and adaptive signaling pathways that promote survival, migration, invasion, and therapeutic resistance. In this study, we investigated the antitumor effects of the plant-derived Kunitz-type protease inhibitor EcTI using both in vitro B16F10-Nex2 melanoma cells and an in vivo murine melanoma model, focusing on adhesion-dependent signaling, autophagy, mitochondrial dysfunction, and regulated cell death. EcTI was efficiently internalized by melanoma cells and showed partial colocalization with lysosomal and mitochondrial compartments, suggesting intracellular trafficking toward these organelles. Treatment reduced cell adhesion to extracellular matrix proteins, particularly fibronectin and laminin, and inhibited migration, invasion, and angiogenic signaling. These effects were associated with modulation of the adhesion-dependent FAK/Src/ERK signaling axis and decreased MMP-9 activity. EcTI also disrupted autophagy, as indicated by accumulation of acidic vesicular organelles, increased LC3-II levels, and modulation of ULK1, Ambra1, and Beclin-1 signaling. In parallel, EcTI induced mitochondrial dysfunction, characterized by loss of mitochondrial membrane potential, intracellular Ca^2+^ dysregulation, and increased reactive oxygen species production. These alterations triggered regulated cell death involving apoptotic and necroptosis-like mechanisms. Importantly, EcTI significantly suppressed tumor growth in vivo without detectable systemic toxicity and modulated inflammatory mediators associated with tumor progression. Overall, these findings demonstrate that EcTI exerts broad antitumor activity by modulating multiple signaling pathways associated with melanoma progression and represents a promising therapeutic candidate for melanoma treatment.

## 1. Introduction

Skin cancer is the most common malignancy worldwide and comprises melanoma and non-melanoma subtypes with distinct biological behaviors and clinical outcomes. Although non-melanoma skin cancers are highly prevalent and rarely metastasize, melanoma accounts for most skin cancer-related deaths due to its aggression and invasive and metastatic potential [1,2]. Despite substantial advances in targeted therapies and immunotherapy, the prognosis of advanced melanoma remains poor because of tumor heterogeneity, phenotypic plasticity, and adaptive resistance mechanisms that promote disease progression and therapeutic failure [3]. Melanoma progression is accompanied by profound metabolic reprogramming that supports survival, invasion, therapeutic resistance, and adaptation to the tumor microenvironment [4]. These challenges highlight the need for novel therapeutic approaches capable of simultaneously targeting multiple pathways involved in metastatic behavior.

Cancer progression is strongly influenced by dynamic interactions between tumor cells and the tumor microenvironment. Among the signaling pathways involved in this process, integrin-mediated adhesion and focal adhesion kinase (FAK) signaling play central roles in regulating cell survival, migration, invasion, and metastatic dissemination. Through interactions with extracellular matrix (ECM) proteins, integrins activate signaling networks that coordinate cytoskeletal remodeling, focal adhesion turnover, cell motility, and resistance to anoikis, thereby promoting tumor progression [5,6,7]. Aberrant activation of FAK signaling has been associated with increased melanoma aggressiveness and metastatic potential, making this pathway an attractive therapeutic target.

The invasive behavior of melanoma cells is also dependent on extracellular proteolytic systems that regulate ECM remodeling and the bioavailability of signaling molecules within the tumor microenvironment. Proteases, including serine proteases and matrix metalloproteinases (MMPs), contribute to extracellular matrix degradation, angiogenesis, tumor invasion, and metastatic dissemination [8,9]. The coordinated interaction between proteolytic activity and adhesion-dependent signaling pathways facilitates tumor adaptation to the surrounding microenvironment and supports multiple hallmarks of cancer [10,11]. Emerging evidence further indicates that adhesion-dependent signaling pathways are functionally linked to intracellular stress responses, including mitochondrial adaptation and survival mechanisms. Consequently, agents capable of modulating both proteolytic and adhesion-related pathways may exert broad antitumor effects.

In addition to extracellular remodeling, mitochondrial homeostasis is a critical determinant of melanoma cell survival and adaptation to environmental stress. Mitochondria function as central regulators of cellular metabolism, redox balance, calcium signaling, and cell death. Mitochondrial dysfunction is commonly associated with loss of mitochondrial membrane potential (ΔΨm), increased reactive oxygen species (ROS) production, dysregulation of intracellular calcium homeostasis, and activation of stress-response pathways [12,13]. Depending on the intensity and duration of stress, these alterations may trigger adaptive mechanisms such as autophagy or activate regulated cell death pathways. Increasing evidence indicates that mitochondrial dysfunction, oxidative stress, autophagy, and cell death signaling are highly interconnected processes that influence melanoma progression and therapeutic responsiveness [14,15,16].

Plant-derived protease inhibitors have emerged as promising multifunctional molecules with potential applications in cancer therapy due to their ability to modulate proteolytic signaling pathways involved in tumor progression, inflammation, angiogenesis, and tissue remodeling [17,18,19,20]. Among these molecules is the *Enterolobium contortisiliquum* trypsin inhibitor (EcTI), a Kunitz-type serine protease inhibitor purified from the seeds of *Enterolobium contortisiliquum*. This inhibitor is a stable protein of approximately 20 kDa characterized by the canonical Kunitz structural fold stabilized by disulfide bridges. EcTI inhibits trypsin with high affinity and also displays inhibitory activity toward plasma kallikrein, plasmin, neutrophil elastase and factor XIIa, thereby modulating inflammatory responses, extracellular matrix remodeling and proteolytic signaling. Previous biochemical and structural studies have demonstrated that EcTI possesses remarkable thermal and pH stability and exhibits multiple biological activities, including anti-inflammatory, antiangiogenic and antitumor effects. Previous studies have demonstrated that EcTI exhibits anti-inflammatory, antiangiogenic, and antitumor activities while displaying low toxicity toward non-tumor cells [21,22,23,24]. In human melanoma cells, EcTI was previously shown to inhibit proliferation, adhesion, migration, and invasion through modulation of Src/FAK-dependent signaling pathways and proteins involved in cell survival and death regulation [22,24]. However, whether these cellular effects translate into effective suppression of melanoma progression in vivo and whether they are associated with mitochondrial dysfunction, oxidative stress, intracellular homeostasis, autophagy-associated pathways, and regulated cell death remain unknown. Therefore, the present study investigated the antitumor activity of EcTI in the highly aggressive B16F10-Nex2 murine melanoma model, integrating analyses of adhesion-dependent signaling, extracellular matrix remodeling, mitochondrial function, oxidative stress, calcium homeostasis, autophagy-associated pathways, regulated cell death, systemic inflammatory mediators, and tumor progression in vivo. This study extends our previous findings by providing in vivo evidence that supports the therapeutic potential of EcTI and offers a more comprehensive understanding of the mechanisms associated with its antitumor activity.

## 2. Results

### 2.1. EcTI Disturbs Melanoma Cell Viability and Proliferation in a Time-Dependent Manner

**Figure 1:**
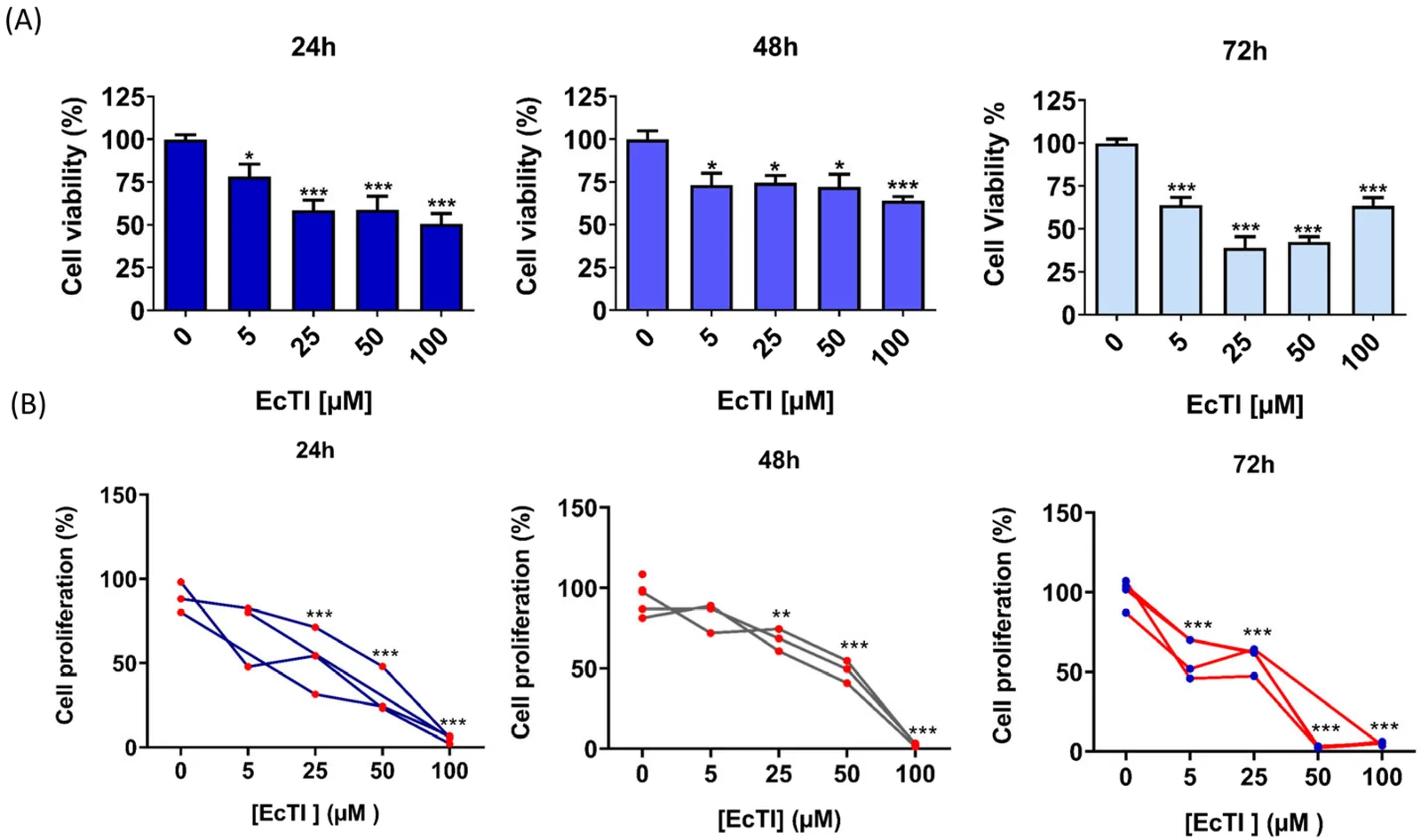
Effects of EcTI on murine melanoma cell viability and proliferation. (**A**) Cell viability after 24, 48 and 72 h of exposure to increasing concentrations of EcTI (0–100 µM). (**B**) Cell proliferation was measured under identical conditions. Data are presented as mean ± SEM. Statistical significance was determined using two-way ANOVA followed by Dunnett’s multiple comparisons test (* *p* < 0.05, ** *p* < 0.01, *** *p* < 0.001).

EcTI significantly decreased the viability of melanoma cells in a dose- and time-dependent manner, with the strongest effect observed after 72 h of treatment. Cell viability was significantly reduced at the highest concentration tested (100 µM) by >80% compared to untreated control cells (*p* < 0.001) (Figure 1A), indicating a strong impairment of cell survival under prolonged exposure.

Consistent with these findings, BrdU incorporation assays showed a marked decrease in DNA synthesis, thereby demonstrating a significant inhibitory effect of EcTI on melanoma cell proliferation (Figure 1B). Taken together, these data demonstrate that EcTI exerts potent antiproliferative effects, particularly after prolonged exposure.

Importantly, as these effects become more evident over time, they are unlikely to entirely explain the early and strong inhibition of migratory and invasive behavior observed in later assays. EcTI may disrupt adhesion-dependent signaling pathways and tumor–microenvironment interactions, likely via modulation of integrin/FAK-mediated signaling, which precedes and may lead to a decrease in cell viability and proliferative capacity.

### 2.2. Melanoma Cells Internalize and Accumulate EcTI in Intracellular Compartments

The intracellular localization of EcTI in B16F10-Nex2 melanoma cells was investigated using fluorescence-based approaches. EcTI was taken up efficiently and demonstrated a punctate cytoplasmic distribution pattern, suggesting accumulation in intracellular vesicular compartments. Co-localization analyses showed a partial overlap of EcTI with lysosomal and mitochondrial markers, indicating intracellular trafficking toward these organelles.

**Figure 2:**
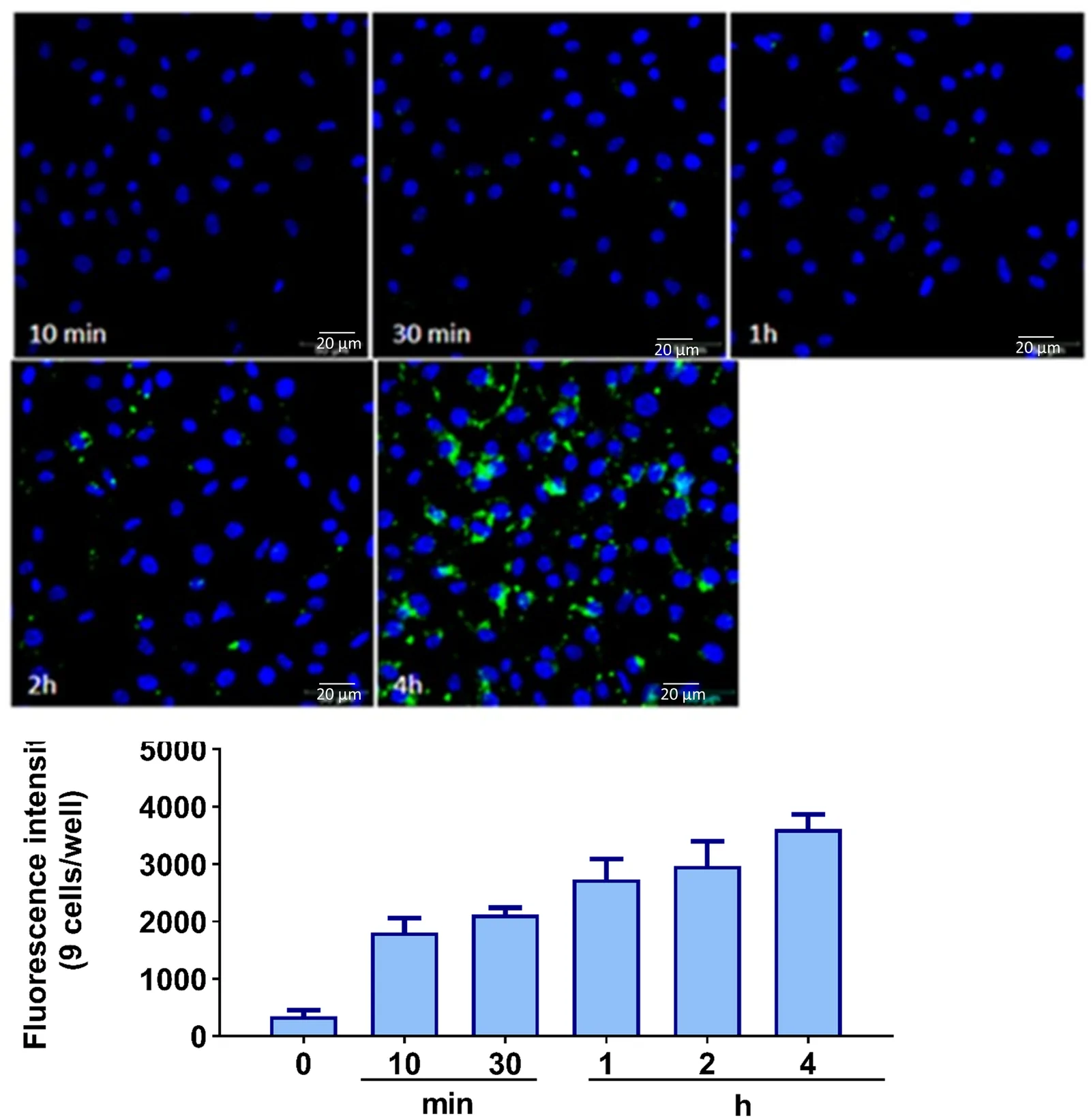
B16F10-Nex2 cells were incubated with EcTI conjugated to Alexa Fluor 488 (100 µM) for 10 min, 30 min, 1 h, 2 h, and 4 h, followed by confocal microscopy analysis. Images show progressive intracellular accumulation of EcTI (green) over time. Nuclei were stained with DAPI (blue). Representative images from three independent experiments are shown.

Time-course experiments showed rapid uptake kinetics with intracellular fluorescence detectable within 10–30 min and increasing up to 4 h after treatment (Figure 2). These results indicate that EcTI is rapidly internalized and remains in the intracellular compartment of melanoma cells.

### 2.3. EcTI Disrupts Melanoma Cell Adhesion, Migration and Invasion

Cell–cell and cell–extracellular matrix (ECM) adhesion are fundamental processes in cancer biology, as they regulate the signaling stimuli mediating intracellular and extracellular responses. To investigate this, the interactions of melanoma cells with major ECM components such as collagen I and IV, laminin, fibronectin and vitronectin were evaluated.

**Figure 3:**
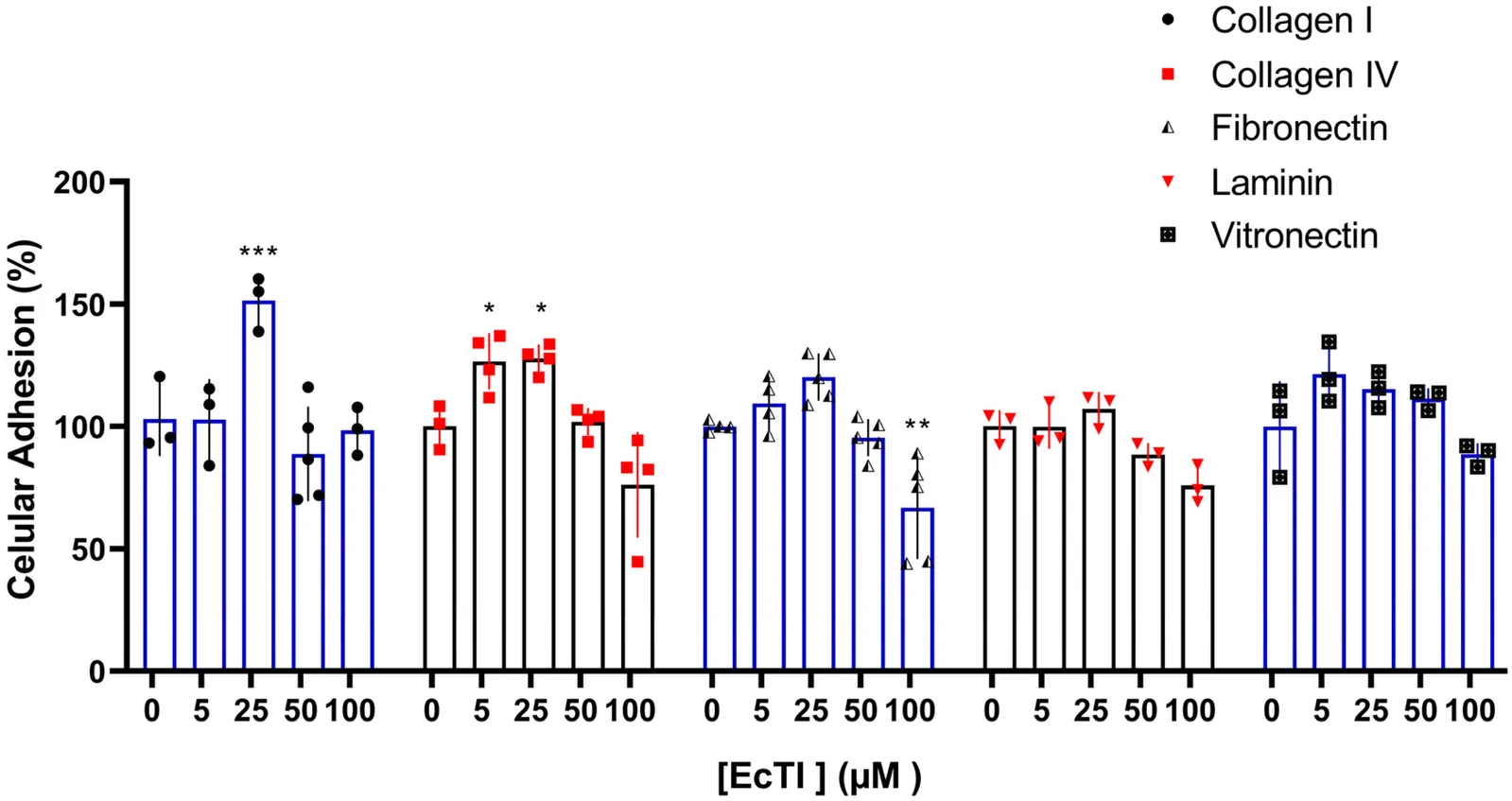
Effect of EcTI on adhesion of cells to extracellular matrix components. Cells were incubated with different concentrations of EcTI (0–100 μM) and the adherence to collagen I, collagen IV, fibronectin, laminin and vitronectin was measured. Data are expressed as mean ± SD of three independent experiments. Statistical significance was determined using two-way ANOVA followed by Dunnett’s multiple comparisons test (* *p* < 0.05, ** *p* < 0.01, *** *p* < 0.001).

EcTI induced a significant decrease in the adhesion capacity of B16F10-Nex2 melanoma cells, particularly in the presence of fibronectin and laminin, where a decrease of about 40% was observed at 100 μM. Our results suggest that EcTI significantly disrupts melanoma cell-ECM interactions, impairing adhesion-dependent signaling pathways (Figure 3). 

**Figure 4:**
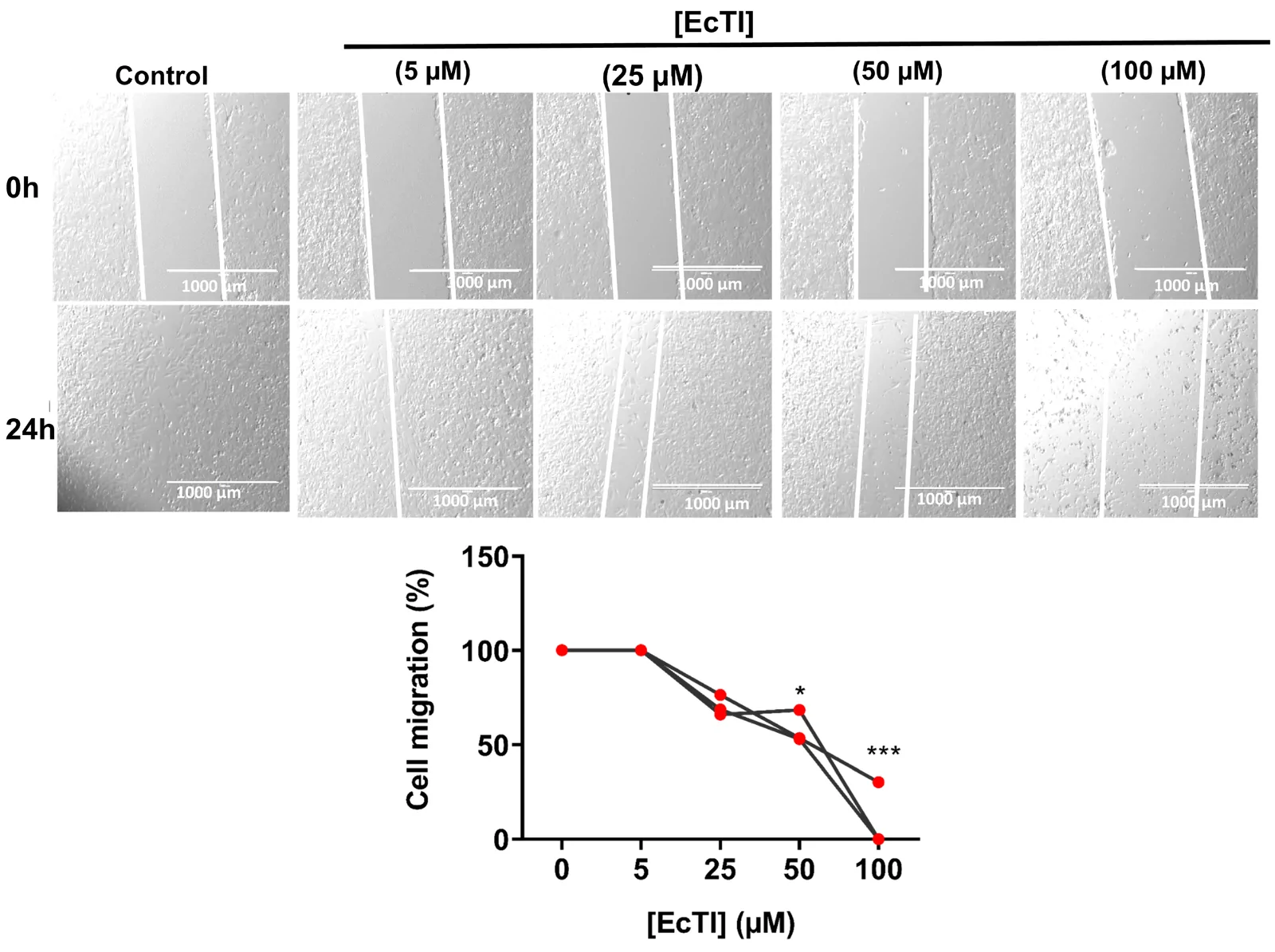
The scratch wound healing assay was used to evaluate cell migration. A linear wound was made with a sterile p200 pipette tip. Detached cells and debris were washed with 1× PBS and adherent cells were cultured in culture medium with EcTI (5, 25, 50 and 100 μM) in a final volume of 400 μL in 24-well plates. Experiments were performed in triplicate. Data are presented as mean ± SEM. Statistical significance was determined using two-way ANOVA followed by Dunnett’s multiple comparisons test (* *p* < 0.05, *** *p* < 0.001).

The effects of EcTI on migratory behavior were subsequently evaluated using wound healing assays. EcTI markedly inhibited melanoma cell migration in a concentration-dependent manner, reducing wound closure by approximately 30%, 40%, and 90% at 25, 50, and 100 µM, respectively (Figure 4).

**Figure 5:**
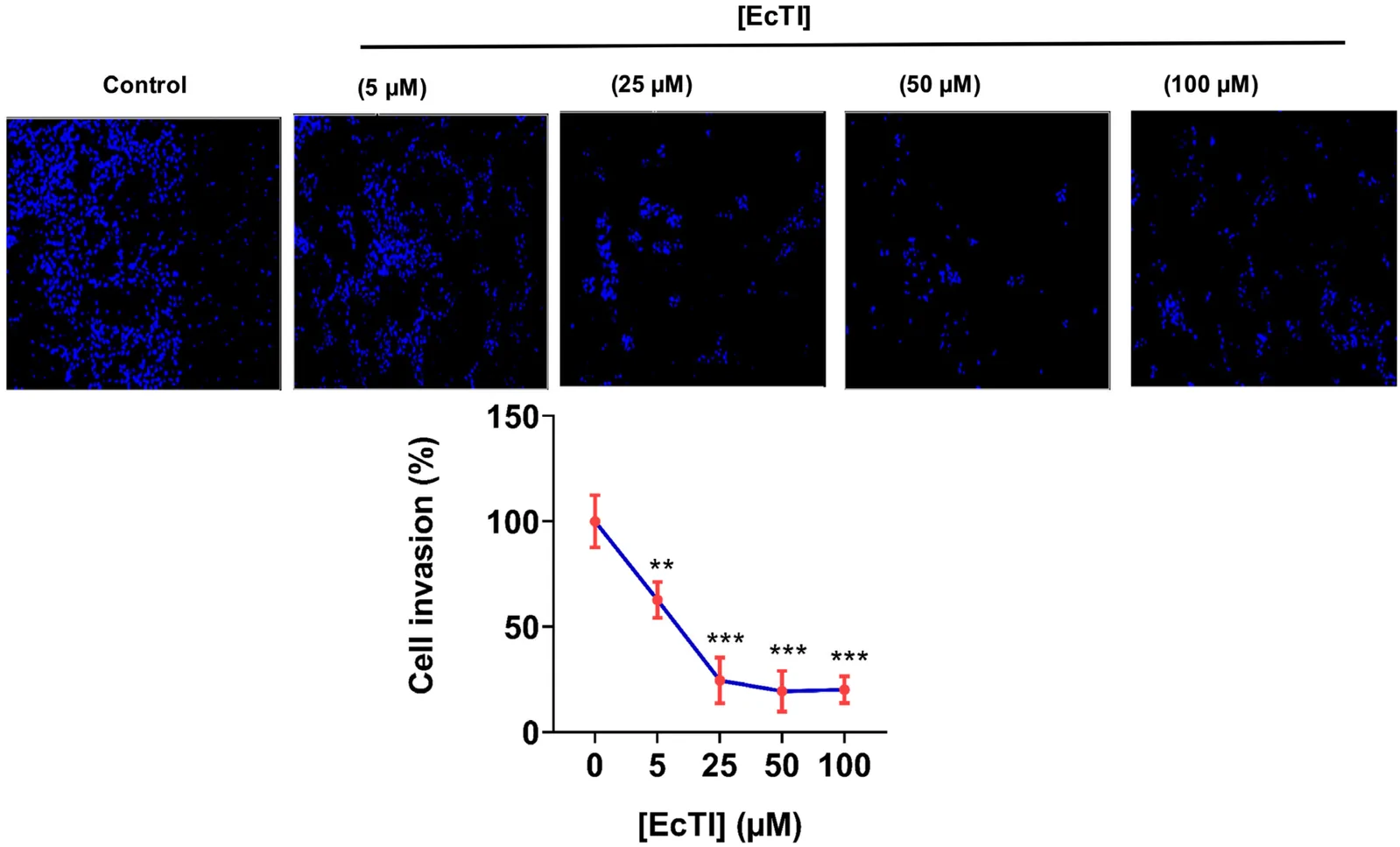
Cell invasion was measured using Matrigel-coated Transwell inserts (8 μm pore size). Adherent cells were cultured with EcTI (5, 25, 50 and 100 μM) in culture medium at a final volume of 250 μL in 24-well plates. Images were taken and the number of invading cells was counted and analyzed. Invasion was calculated as a percentage of invading cells compared to the control (untreated cells). Experiments were performed in triplicate, and statistical significance was determined using two-way ANOVA followed by Dunnett’s multiple comparisons test (** *p* < 0.01, *** *p* < 0.001).

Similarly, matrigel invasion assays demonstrated a significant reduction in the number of invading cells following treatment with 50 and 100 µM EcTI (*p* < 0.01–0.001) (Figure 5). Importantly, viability controls performed under the same experimental conditions indicated that the inhibitory effects on migration and invasion were not exclusively attributable to cytotoxicity.

Together, these findings indicate that EcTI interferes with cellular mechanisms associated with adhesion, motility, and invasive behavior in melanoma cells.

### 2.4. EcTI Suppresses Extracellular Matrix Remodeling and Angiogenic Signaling

**Figure 6:**
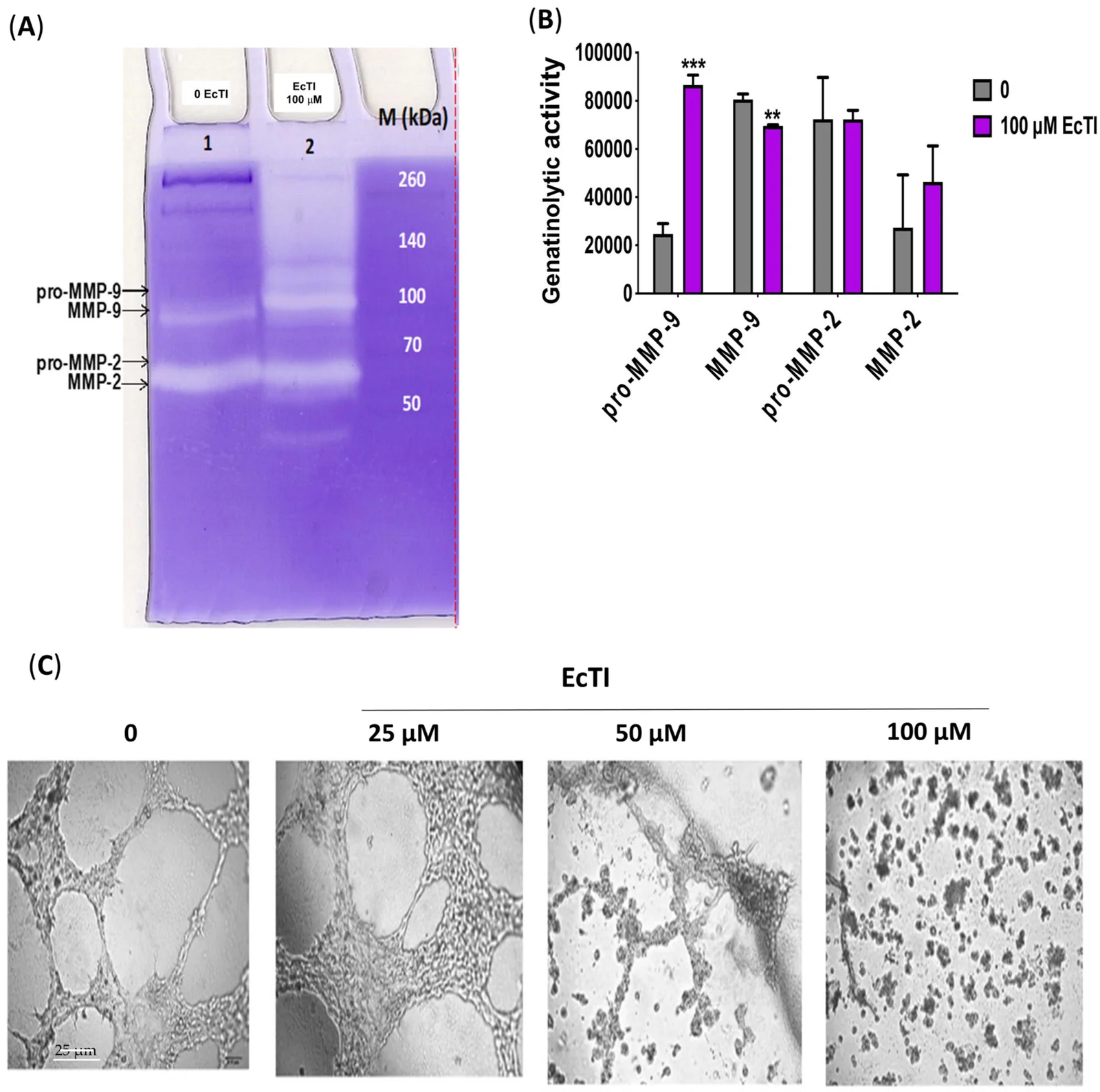
EcTI inhibits metalloproteinase activity and blocks tumor-derived angiogenic signaling in B16F10-Nex2 melanoma cells. (**A**) Representative gelatin zymography of conditioned medium from B16F10-Nex2 cells treated with 100 μM EcTI. Lane 1: untreated control; Lane 2: EcTI-treated cells; M: molecular weight marker (Spectra™ Multicolor Broad Range Protein Ladder, Thermo Fisher Scientific, Waltham, MA, USA). The positions of pro-MMP-9 (92 kDa), MMP-9 (84 kDa), pro-MMP-2 (72 kDa), and MMP-2 (62 kDa) are indicated. (**B**) Densitometric quantification of gelatinolytic activity was performed using ImageJ after LUT inversion. Data are presented as mean ± SD of three independent experiments. Statistical significance was determined by one-way ANOVA followed by Tukey’s multiple comparisons test (** *p* < 0.01, *** *p* < 0.001). (**C**) Representative images of HUVEC tube formation cultured with conditioned medium from B16F10-Nex2 cells treated with 0, 25, 50, or 100 μM EcTI. Conditioned medium from EcTI-treated melanoma cells reduced endothelial tube formation in a concentration-dependent manner. Scale bar = 25 μm.

To investigate whether EcTI modulates proteolytic mechanisms associated with tumor invasion, the activity of matrix metalloproteinases (MMPs) was evaluated by gelatin zymography. Gelatinolytic bands corresponding to pro-MMP-2/active MMP-2 and pro-MMP-9/active MMP-9 were detected at the expected molecular weights (Figure 6A).

EcTI treatment significantly reduced active MMP-9 levels while increasing accumulation of pro-MMP-9 in B16F10-Nex2 cells. Quantitative analysis demonstrated an approximately fourfold increase in pro-MMP-9 levels accompanied by a reduction in active MMP-9 following treatment with 100 µM EcTI (Figure 6B). These results are in agreement with the decreased invasive ability of EcTI-treated cells and support a role for EcTI in the modulation of protease-dependent mechanisms involved in tumor progression.

Considering the close association between extracellular matrix remodeling and angiogenesis, the effects of EcTI on tumor-derived angiogenic signaling were further investigated using conditioned media from treated melanoma cells in HUVEC tube formation assays. Conditioned media obtained from EcTI-treated cells significantly impaired endothelial tube formation in a concentration-dependent manner (Figure 6C). At 50 and 100 µM, endothelial organization was markedly disrupted, with near-complete inhibition of closed capillary-like network formation at the highest concentration tested.

These findings provide evidence that EcTI modulates extracellular matrix-associated signaling and indirectly impairs angiogenic responses mediated by tumor-derived factors.

### 2.5. EcTI Inhibits Focal Adhesion Signaling Pathways and Modulates Autophagy-Related Proteins

**Figure 7:**
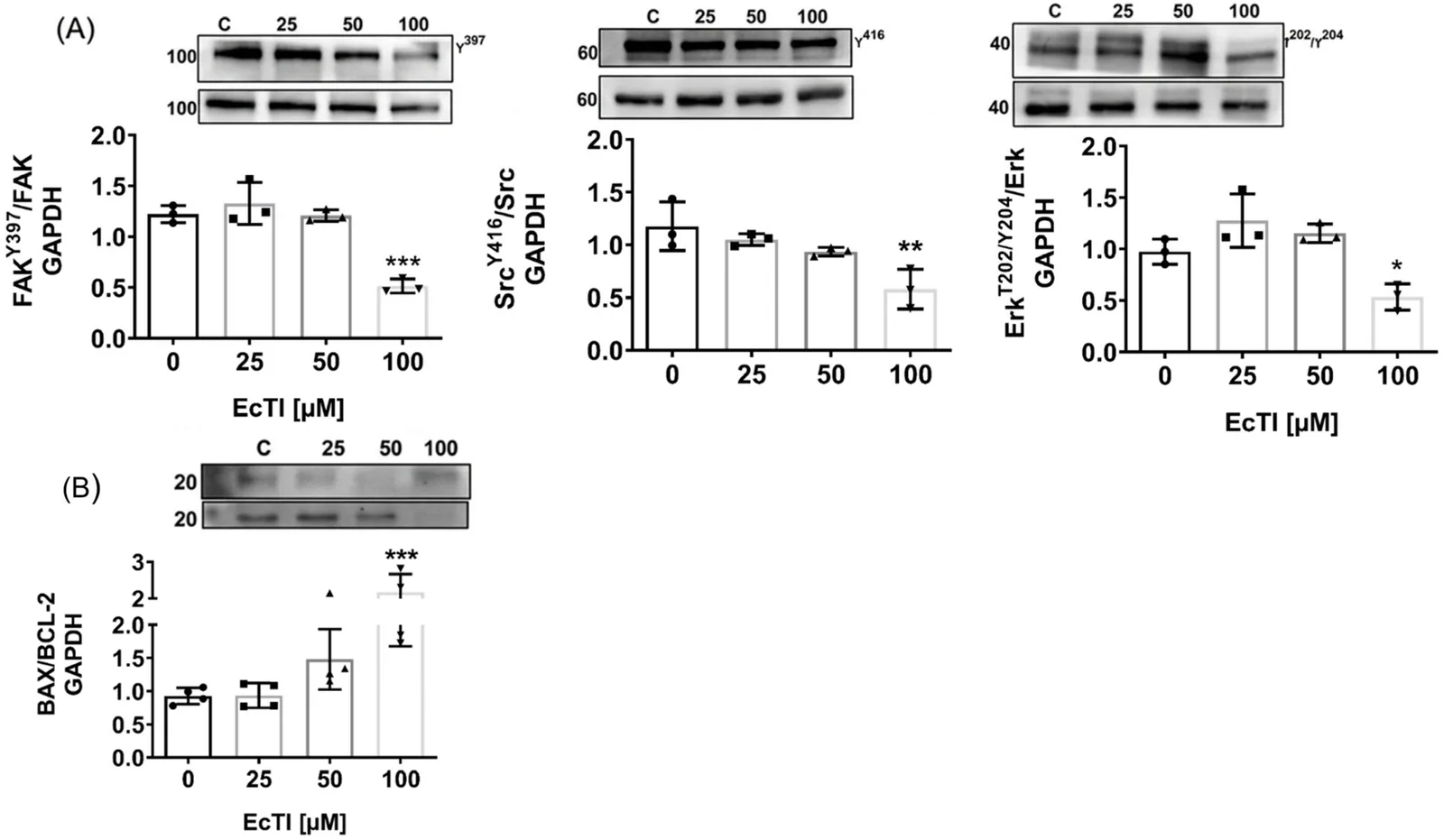

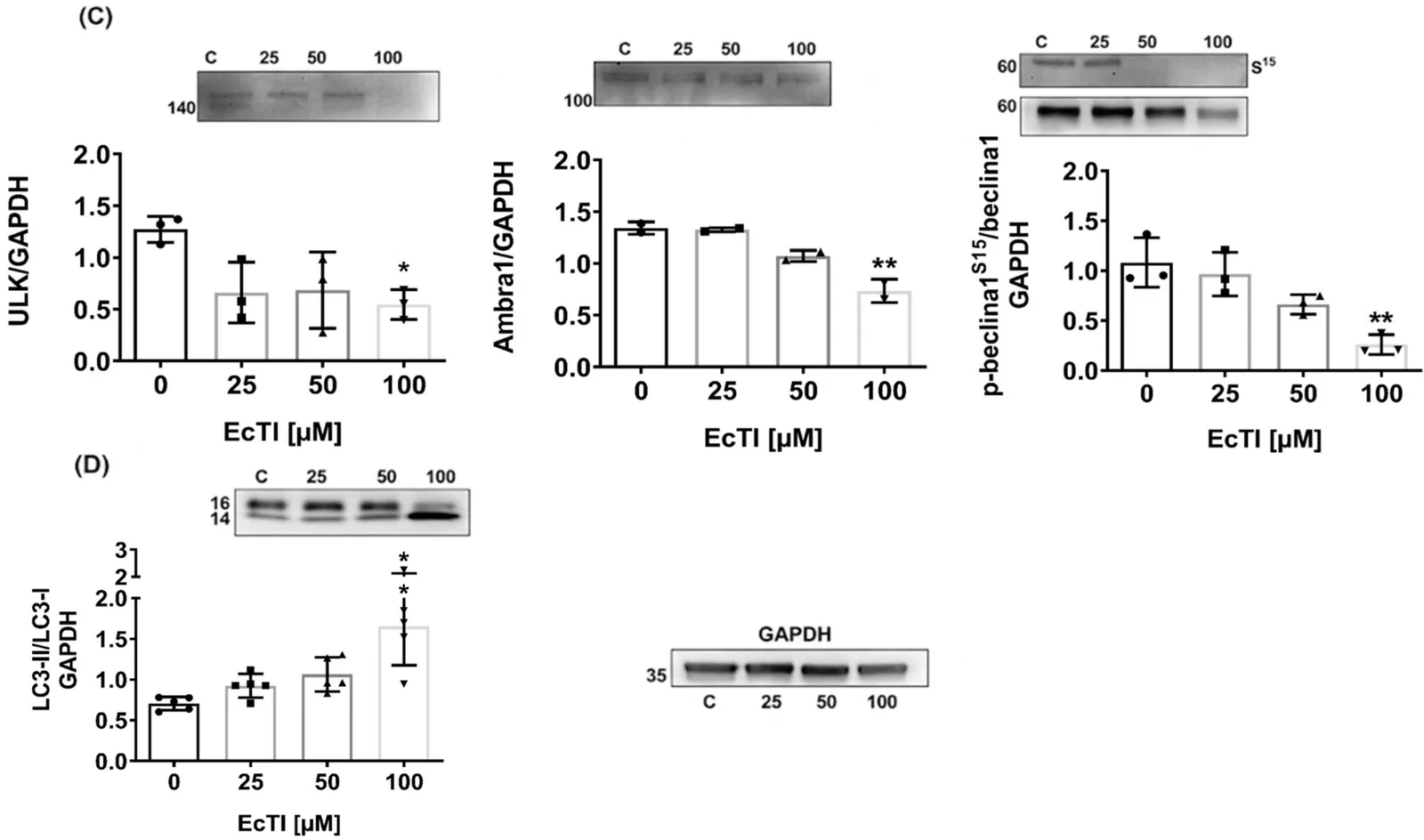
Cells were treated with EcTI (25-100 μM) for 24 h. (**A**) Representative immunoblots and densitometric analyses of p-FAK/FAK, p-Src/Src, and p-ERK/ERK. (**B**) Bax and Bcl-2 expression levels. (**C**) ULK1, AMBRA1, and phospho-Beclin-1 expression. (**D**) LC3-I and LC3-II expression and the LC3-II/LC3-I ratio. Data are expressed as mean ± SD from three independent experiments. Statistical significance was determined by one-way ANOVA followed by Tukey’s post hoc test (* *p* < 0.05, ** *p* < 0.01, *** *p* < 0.001 versus control). All proteins were analyzed from the same experimental set, and expression levels were normalized to GAPDH. Shapes represent different amounts of the horizontal axis.

To explore the molecular basis of the impaired migratory phenotype and the intracellular stress responses induced by EcTI, we assessed proteins involved in focal adhesion signaling, survival pathways, apoptosis regulation, and autophagy-related processes (Figure 7). EcTI treatment (25–100 µM, 24 h) significantly reduced phosphorylation of FAK (Tyr397) and Src (Tyr416), indicating inhibition of focal adhesion signaling. This effect was associated with reduced ERK phosphorylation, suggesting suppression of downstream MAPK pathways involved in proliferation and cell survival (Figure 7A).

At the same time, EcTI increased the Bax/Bcl-2 ratio, indicative of a pro-apoptotic intracellular environment and mitochondrial dysfunction (Figure 7B). In addition, EcTI modulated key autophagy-related proteins. Treatment reduced ULK1 and Ambra1 expression and decreased Beclin-1 phosphorylation (Ser15), while markedly increasing the LC3-II/LC3-I ratio (4-fold at 100 µM), indicating substantial alterations in autophagy-associated pathways (Figure 7C,D).

Altogether, these findings demonstrate that EcTI inhibits the FAK/Src/ERK signaling axis while simultaneously modulating apoptotic and autophagy-related regulators, thereby compromising adhesion, migration, survival, and intracellular stress adaptation mechanisms in melanoma cells.

### 2.6. EcTI Induces Accumulation of Acidic Vesicular Organelles

**Figure 8:**
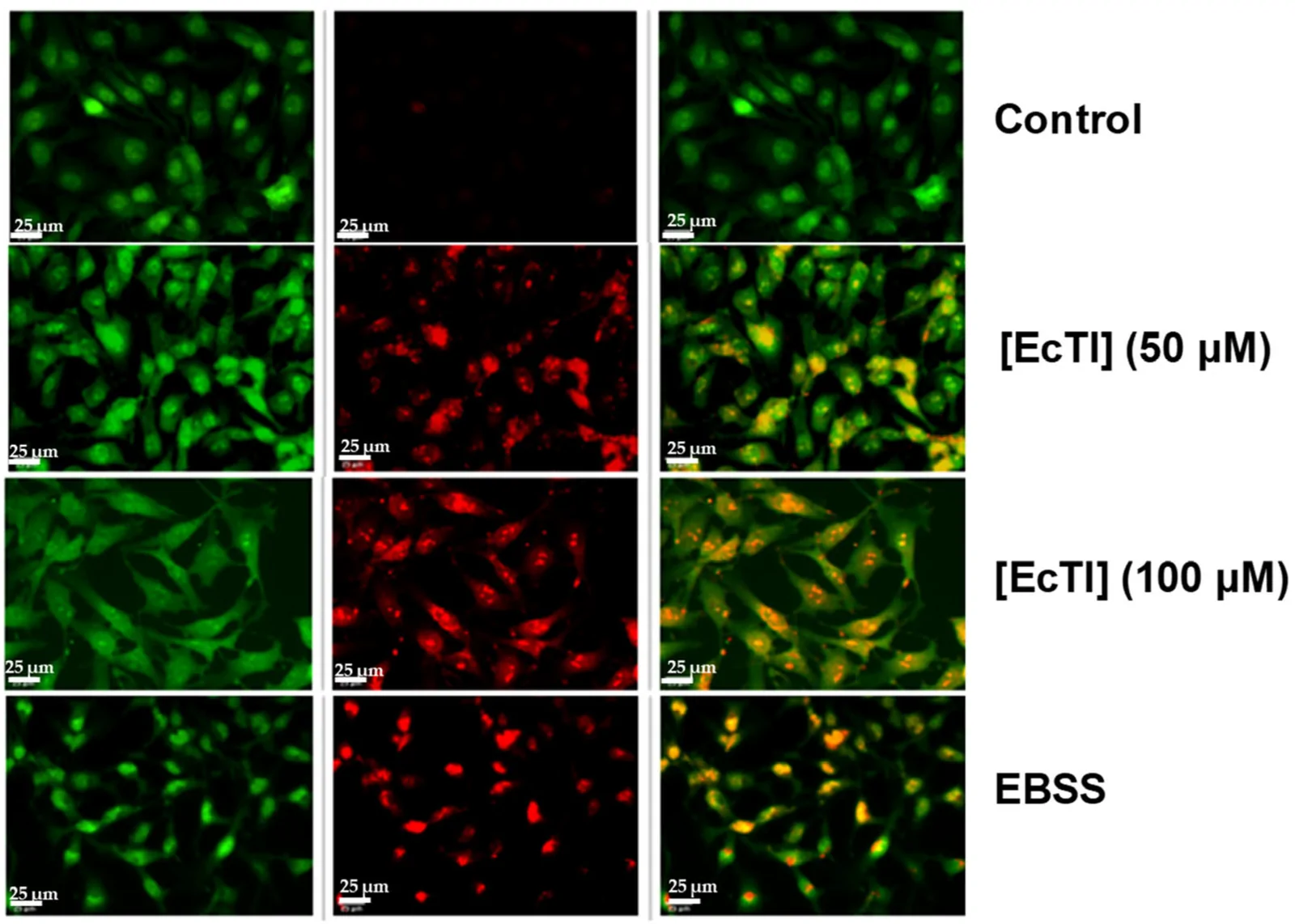
Formation of acidic vesicular organelles (AVOs) was evaluated by acridine orange staining in B16F10-Nex2 melanoma cells treated with EcTI (50 and 100 µM) for 24 h. Nutrient starvation induced by incubation in Earle’s Balanced Salt Solution (EBSS) for 4 h was used as a positive control for autophagy-related vesicular acidification. Representative fluorescence images are shown.

To further investigate whether the molecular alterations observed in autophagy-related proteins were accompanied by changes in vesicular acidification, we evaluated the formation of acidic vesicular organelles (AVOs) using acridine orange staining. EcTI treatment (50 and 100 µM) significantly increased AVO accumulation compared with untreated cells (Figure 8), consistent with expansion of the acidic vesicular compartment.

The increase in AVO formation together with the modulation of LC3, ULK1, Ambra1, and Beclin-1 suggests that EcTI induces autophagy dysregulation. 

### 2.7. EcTI Triggers Mitochondrial Dysfunction, Ca^2+^ Dysregulation and Oxidative Stress

**Figure 9:**
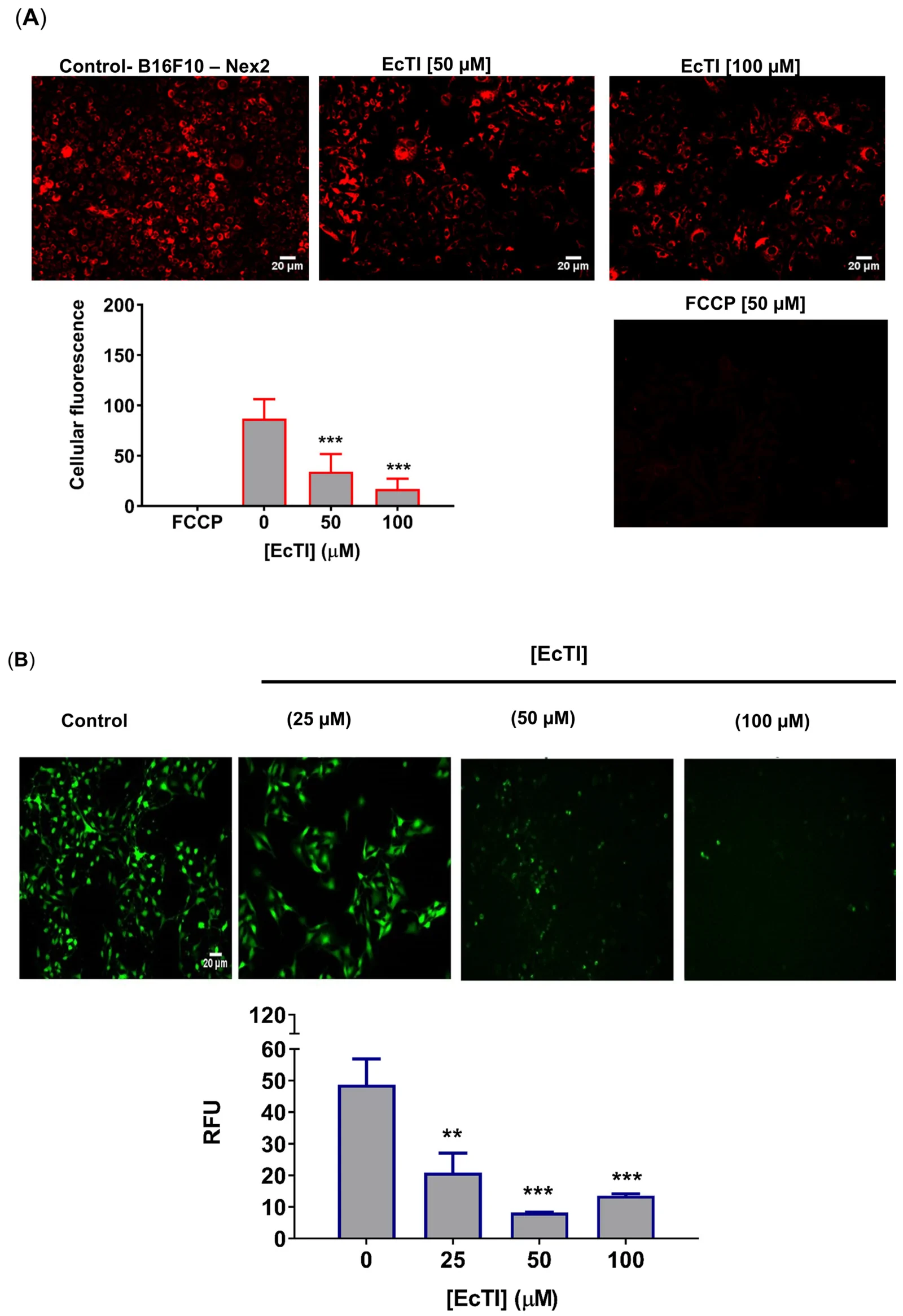

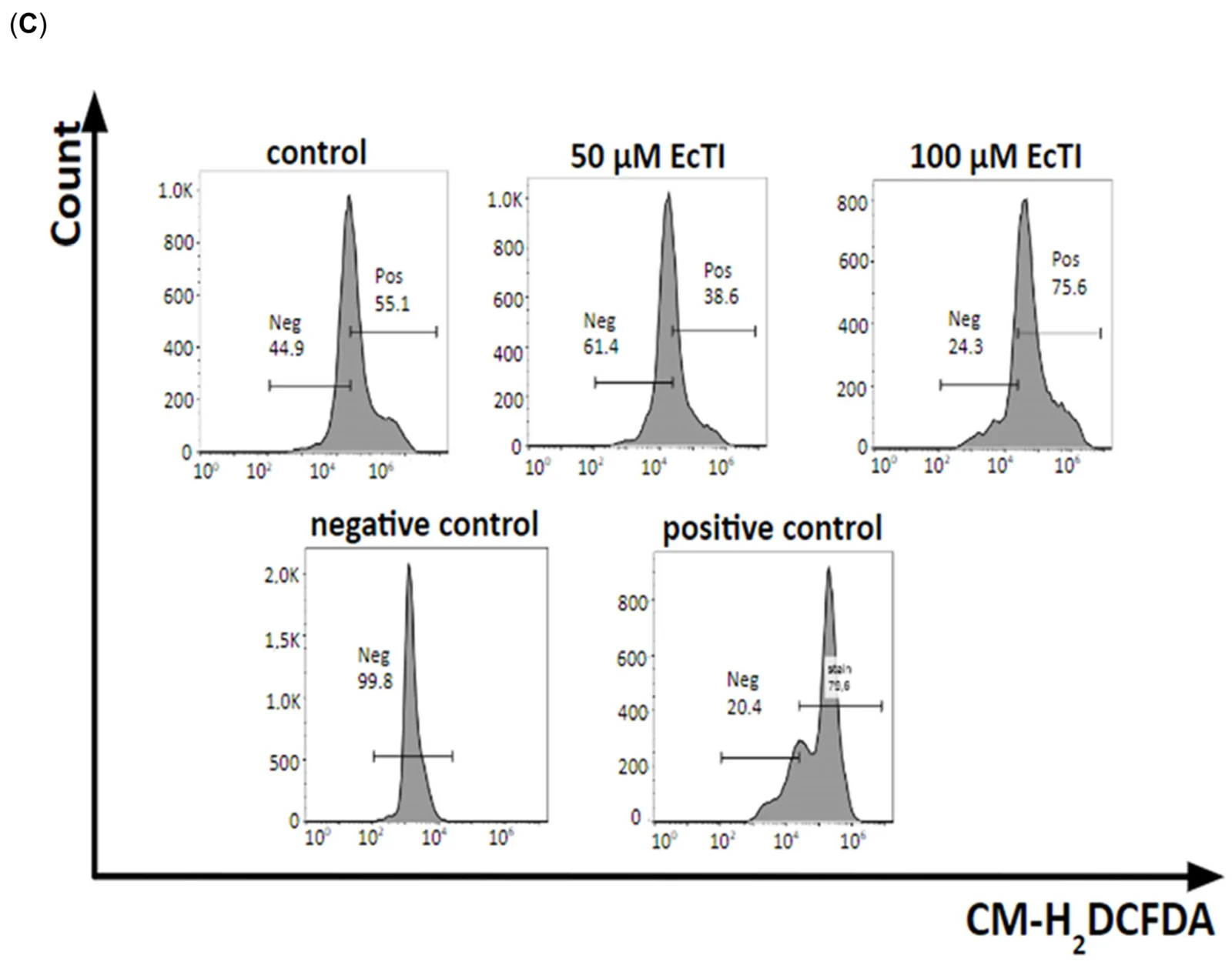
(**A**) Mitochondrial membrane potential (ΔΨm) was assessed by TMRE staining after 24 h of treatment with EcTI (5–100 µM). FCCP (50 µM) was used as a positive control for mitochondrial depolarization. (**B**) Intracellular calcium levels were measured using the Fluo-4 AM fluorescent probe. (**C**) Intracellular reactive oxygen species (ROS) levels were determined using DCFDA staining. Data represent mean ± SEM from three independent experiments, with at least 30,000 cells analyzed per condition. Statistical significance was determined by two-way ANOVA followed by Dunnett’s multiple comparisons test (** *p* < 0.01, *** *p* < 0.001).

Next, we evaluated the effects of EcTI on mitochondrial function in view of its preferential mitochondrial localization. Treatment with EcTI significantly reduced mitochondrial membrane potential (ΔΨm) at 50 and 100 μM, respectively (63 and 84% decrease; *p* < 0.001) (Figure 9A), indicating mitochondrial depolarization.

Simultaneously, EcTI disturbed intracellular calcium homeostasis leading to a significant increase in cytosolic Ca^2+^ levels (Figure 9B). Moreover, EcTI caused a significant increase in intracellular ROS levels, reaching 75.6% at 100 µM after 24 h (*p* < 0.001) (Figure 9C). Together, these results show that EcTI induces mitochondrial dysfunction related to Ca^2+^ imbalance and oxidative stress, hallmarks of the mitochondrial stress-mediated signaling pathways.

### 2.8. EcTI Promotes Multimodal Regulated Cell Death

**Figure 10:**
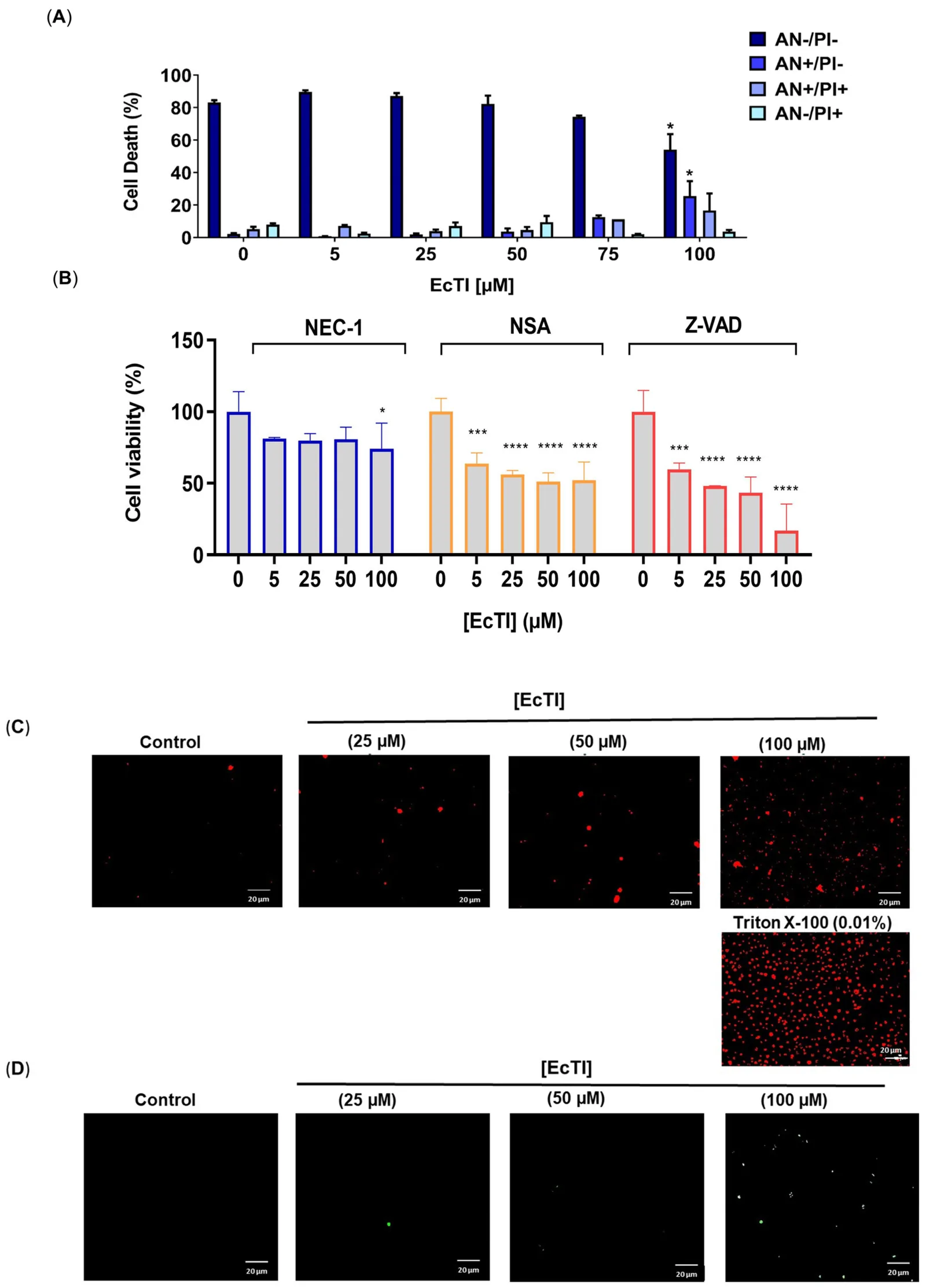

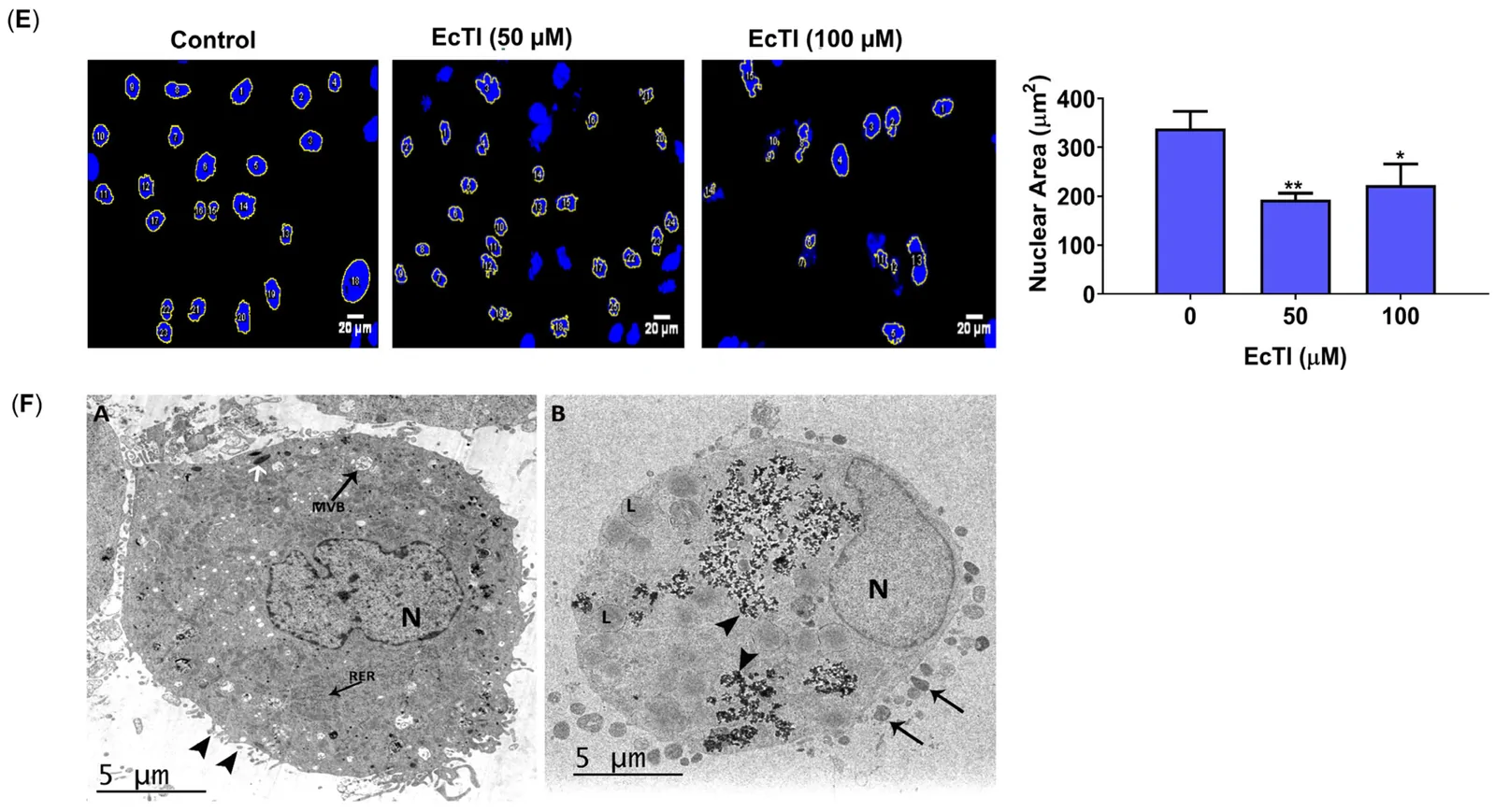
(**A**) Annexin V/PI staining showing the percentage of viable, early apoptotic, late apoptotic, and necrotic cells following EcTI treatment. (**B**) Cell viability after treatment with EcTI in the presence or absence of the apoptosis inhibitor Z-VAD-FMK or the necroptosis inhibitors necrostatin-1 (Nec-1) and necrosulfonamide (NSA). (**C**) Propidium iodide (PI) incorporation assay showing plasma membrane permeability after EcTI treatment. Triton X-100 (0.01%) was used as a positive control. (**D**) Caspase-3/7 activity following EcTI treatment. (**E**) Representative Hoechst 33342 staining and quantification of nuclear condensation after EcTI treatment. (**F**) Representative transmission electron microscopy (TEM) images of untreated control cells (**A**) and EcTI-treated cells (**B**), showing ultrastructural alterations including nuclear marginalization, plasma membrane blebbing, lipid droplet accumulation, and electron-dense intracellular material. Scale bars are indicated. Data are presented as mean ± SEM from at least three independent experiments. Statistical significance is indicated in the graphs (* *p* < 0.05, ** *p* < 0.01, *** *p* < 0.001, **** *p* < 0.0001).

To investigate the activation of regulated cell death pathways, annexin V/propidium iodide staining was performed to detect apoptotic cells. EcTI treatment increased the apoptotic cell population in a concentration-dependent manner (Figure 10A). Pharmacological inhibition assays revealed that treatment with the pan-caspase inhibitor Z-VAD-FMK partially restored cell viability, further supporting the involvement of caspase-dependent apoptosis. In parallel, treatment with necrostatin-1 and necrosulfonamide also partially rescued cell viability (Figure 10B), suggesting the participation of necroptotic mechanisms.

EcTI treatment increased propidium iodide uptake (Figure 10C) and caspase-3/7 activation (Figure 10D), indicating loss of membrane integrity and activation of apoptotic signaling. These findings are consistent with the increased Bax/Bcl-2 ratio observed after EcTI treatment, supporting activation of mitochondrial apoptotic pathways. In addition, live-cell Hoechst 33342 staining revealed significant nuclear condensation and shrinkage following treatment with 100 μM EcTI, with a marked reduction in nuclear area in B16F10-Nex2 cells (Figure 10E).

Ultrastructural analysis by transmission electron microscopy revealed pronounced morphological alterations in treated cells, including nuclear marginalization, lipid droplet accumulation, deposition of electron-dense material (presumably internalized EcTI), membrane blebbing, and cytoplasmic vacuolization (Figure 10F). In contrast, control cells exhibited preserved cellular morphology and organelle organization.

Together, these data indicate that EcTI induces multiple forms of regulated cell death in melanoma cells, involving both apoptotic and necroptotic features.

### 2.9. EcTI Suppresses Tumor Progression In Vivo

The EcTI antitumor activity was further evaluated in vivo using the B16F10-Nex2 melanoma model.

**Figure 11:**
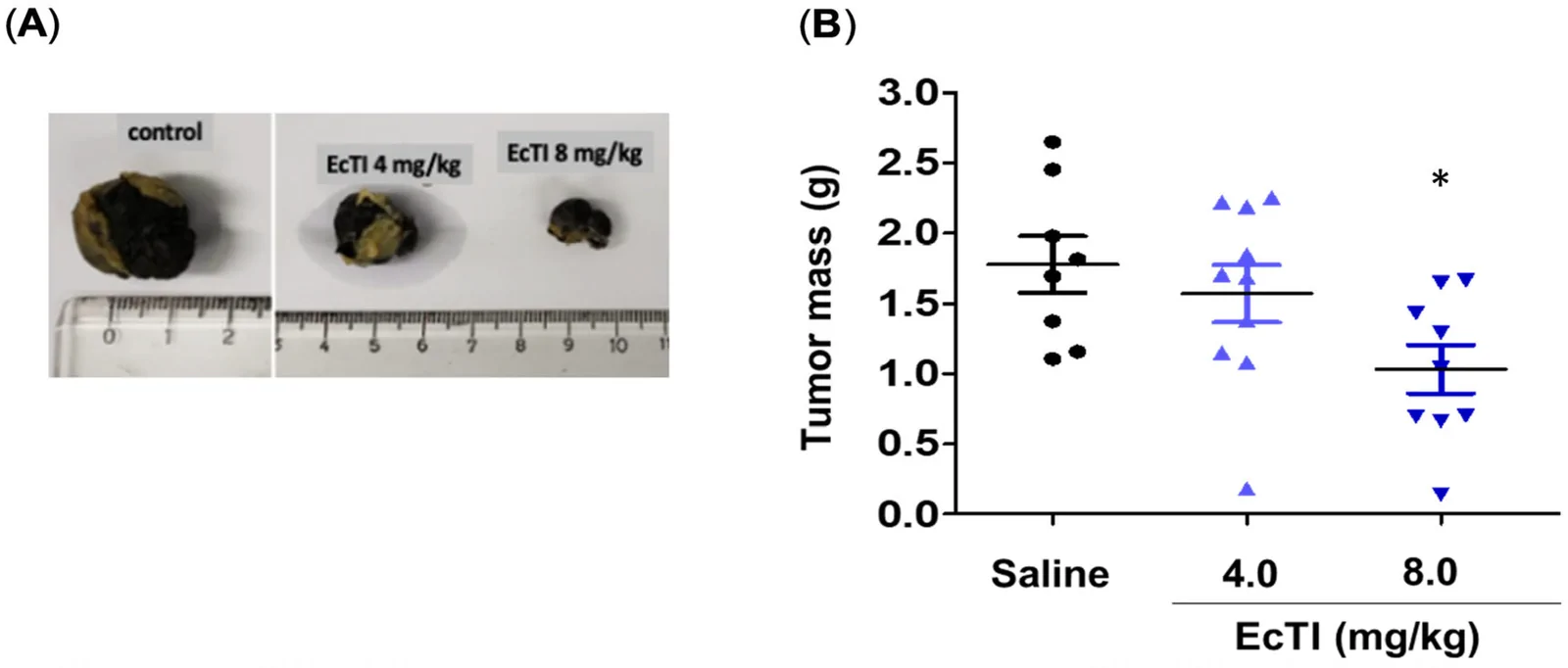
Effect of EcTI treatment on tumor burden in B16F10-Nex2 melanoma-bearing mice. B16F10-Nex2 melanoma cells were subcutaneously inoculated into mice. Following tumor establishment, ani-mals were treated daily with intraperitoneal injections of EcTI (4 or 8 mg/kg), while control animals received saline. (**A**) Representative images of excised tumors from saline- and EcTI-treated animals at the experimental endpoint. (**B**) Tumor mass measured after tumor excision at the experimental endpoint. Individual symbols represent individual animals: black circles (●), saline group; blue up-ward triangles (▲), EcTI 4 mg/kg group; and blue downward triangles (▼), EcTI 8 mg/kg group. Horizontal lines indicate the mean, and vertical error bars represent SEM. Statistical analysis was performed using one-way ANOVA followed by Tukey’s multiple-comparisons test * *p* < 0.05 vs. vehicle).

Animals treated with EcTI (8 mg/kg) showed a significant decrease in tumor progression, as shown by the reduction in tumor volume throughout the study and tumor mass at the end of the experiment, compared with untreated tumor-bearing animals (Figure 11A,B).

Importantly, there was no significant difference in the body weight of EcTI-treated animals during the experimental period, indicating the lack of overt systemic toxicity under the conditions tested.

The histological examination of tumor tissues did not show extensive classical morphological features of acute cell death.

### 2.10. EcTI Modulates Systemic Inflammatory Mediators in Mice Bearing Tumors

Cytokine and chemokine profiles were analyzed in tumor-bearing mice to study systemic effects of EcTI treatment.

**Figure 12:**
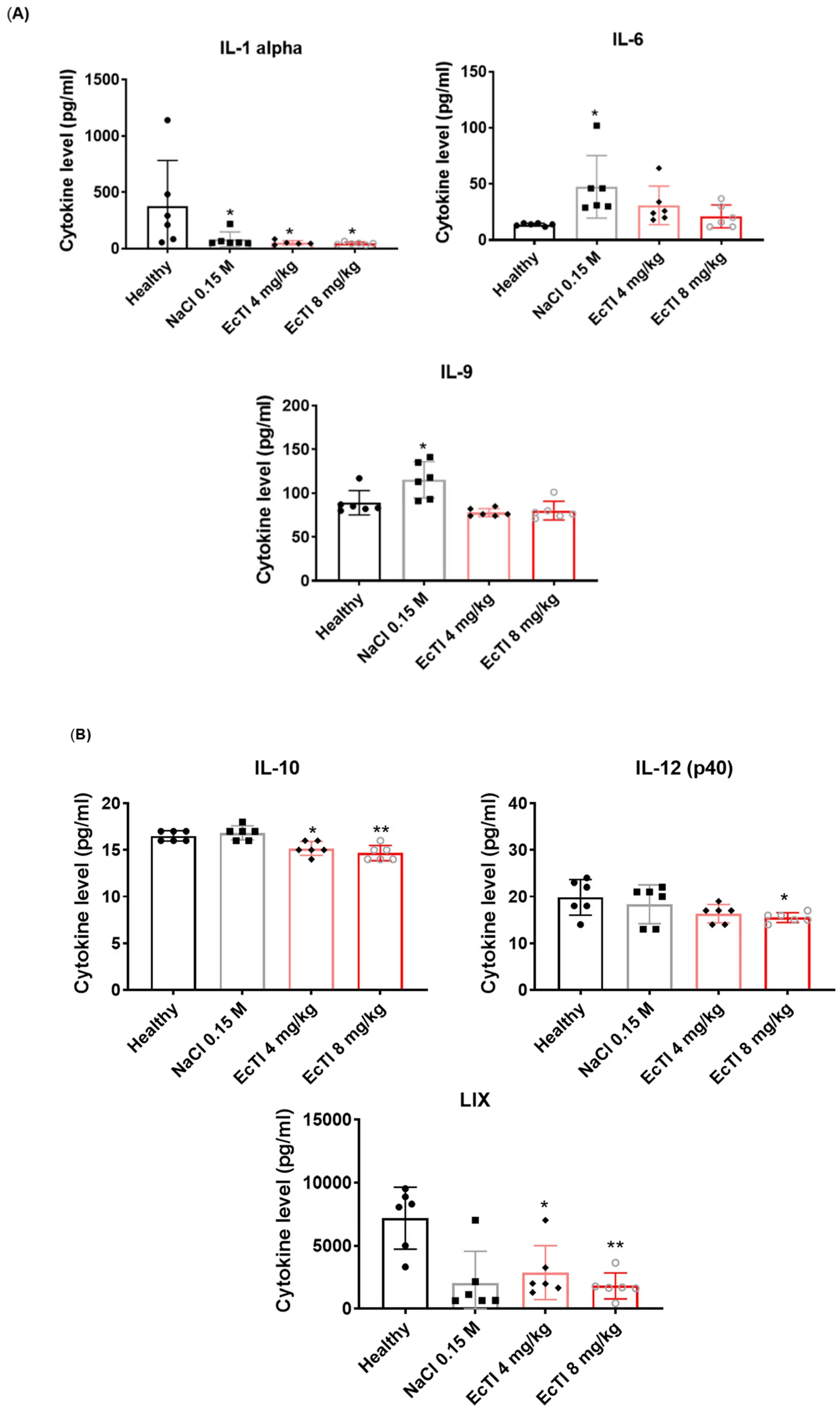

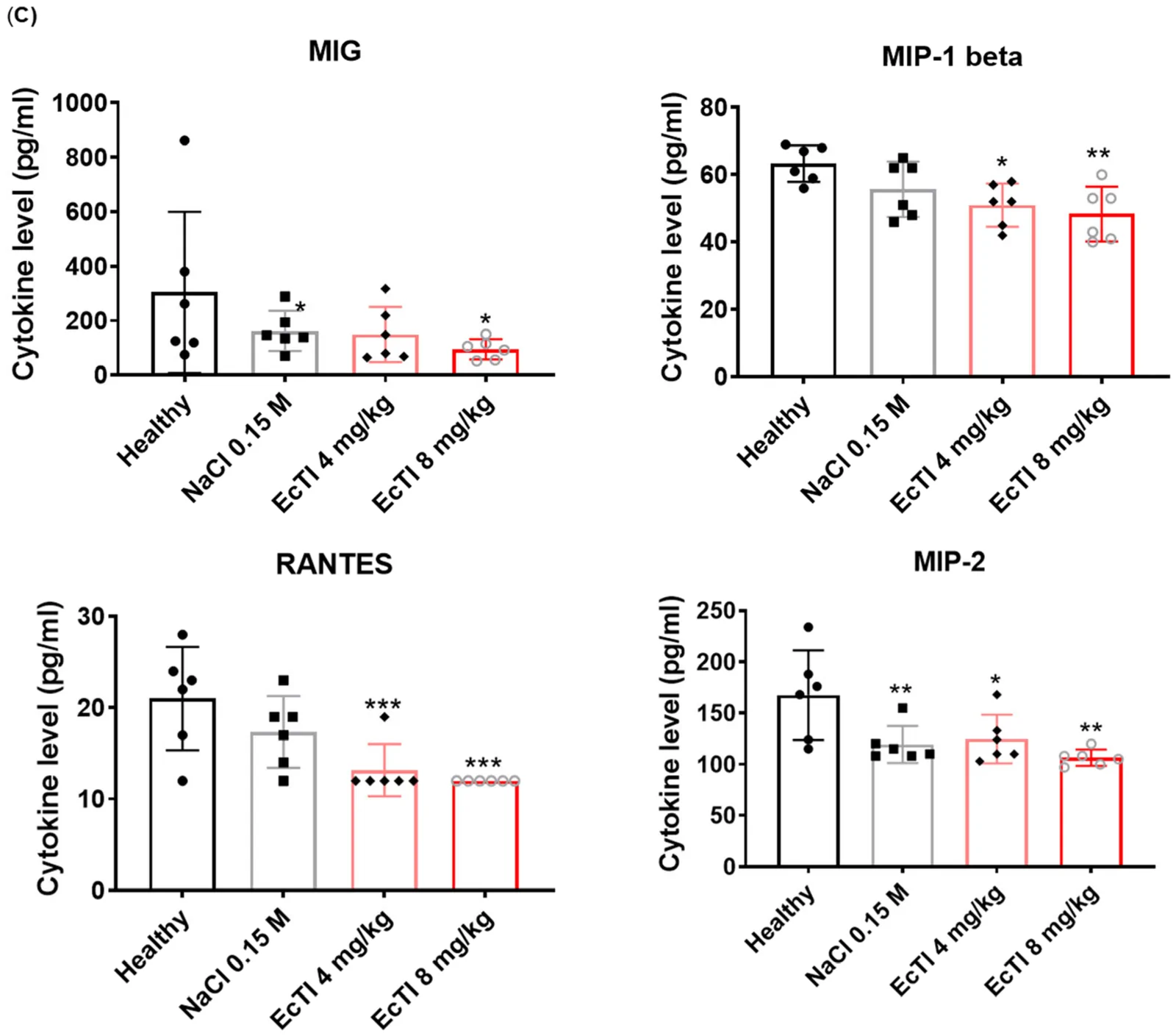
(**A**) Plasma levels of the pro-inflammatory cytokines IL-1α, IL-6, and IL-9. (**B**) Plasma levels of the immune regulatory mediators IL-10, IL-12 (p40), and LIX. (**C**) Plasma levels of the chemokines MIP-2, MIP-1β, MIG, and RANTES. Cytokine and chemokine concentrations were determined by multiplex immunoassay. Bars represent group mean ± SEM (n = 6 mice per group), symbols (●, ■, ♦) denote individual animals/samples. Bars represent mean values and error bars indicate SEM. Statistical significance was determined by one-way ANOVA followed by Tukey’s multiple comparisons test (* *p* < 0.05, ** *p* < 0.01, *** *p* < 0.001).

Tumor-bearing animals showed high levels of pro-inflammatory cytokines IL-1α, IL-6 and IL-9. These mediators were significantly reduced by EcTI treatment (Figure 12A), implying a modulation of systemic inflammatory responses.

EcTI also significantly decreased IL-10 levels at the two doses tested and IL-12 (p40) levels at 8 mg/kg, but significantly increased LIX levels at 4 mg/kg (Figure 12B).

Chemokine analysis also showed changes in inflammatory mediators involved in leukocyte recruitment. The levels of MIG and RANTES were reduced after EcTI treatment. However, tumor-bearing animals showed reduced levels of MIP-2 and MIP-1β (Figure 12C). These data suggest that EcTI modulates systemic inflammatory and chemotactic mediators related to melanoma progression.

## 3. Discussion

The present study demonstrates that the plant-derived Kunitz-type protease inhibitor EcTI exerts a broad antitumor effect against melanoma by targeting multiple adaptive pathways involved in tumor progression. Our findings extend previous observations obtained in human melanoma cells by demonstrating that the cellular effects of EcTI are accompanied by significant inhibition of tumor progression in an in vivo melanoma model. Collectively, these findings support that EcTI modulates multiple biological processes associated with melanoma progression, affecting both intrinsic tumor signaling and tumor–microenvironment interactions.

Cancer dissemination strongly depends on dynamic communication between tumor cells and the extracellular matrix, involving integrin signaling, focal adhesion remodeling, cytoskeletal plasticity, and proteolytic activity [6,10,11,25]. In this context, the early impairment of adhesion, migration, and invasion observed after EcTI treatment strongly suggests modulation of integrin-mediated adhesive pathways. Previous studies from our group showed that EcTI inhibits Src/FAK signaling and decreases melanoma cell adhesion and invasion [24], which supports our current findings.

Focal adhesion kinase (FAK) is a central signaling hub that integrates extracellular matrix sensing with pathways that control survival, migration, proliferation, and mechanotransduction activities [5,26]. Integrin/FAK signaling dynamically controls focal adhesion turnover and cytoskeletal organization via Rho GTPase-dependent pathways [27,28]. Thus, the decreased adhesion and motility observed here likely reflect an impaired ability of melanoma cells to coordinate cytoskeletal remodeling and extracellular matrix interactions required for invasive behavior. Anchorage-dependent survival is key for tumor progression; therefore, continuous blockade of this axis may ultimately reduce cell proliferation and viability through loss of survival signaling and activation of anoikis-related pathways [29,30].

Critically, EcTI reduced migration and invasion before inducing substantial loss of viability, suggesting that impairment of tumor plasticity represents an early event in its mechanism of action. This temporal sequence is particularly relevant in melanoma, where metastatic dissemination depends on dynamic interactions with the extracellular matrix and precedes extensive tumor expansion. Therefore, EcTI may interfere with the acquisition of invasive phenotypes before triggering irreversible cell damage, a characteristic shared by agents that modulate focal adhesion signaling networks [25,31]. Taken together, these findings suggest that modulation of adhesion-dependent processes constitutes an early component of the cellular response to EcTI, preceding the activation of intracellular stress responses and regulated cell death pathways.

The present results further demonstrate the profound effect of EcTI on intracellular homeostasis. The efficient internalization of EcTI and its localization to lysosomal and mitochondrial compartments indicate that this inhibitor reaches intracellular compartments associated with proteolytic and metabolic regulation. Proteases are not only involved in the remodeling of the extracellular matrix but also in protein turnover within the cell, regulation of signaling, organelle homeostasis, and stress adaptation [8,9]. Interference with protease-dependent pathways may therefore contribute to the broad cellular dysfunction induced by EcTI, linking extracellular protease inhibition to intracellular stress responses.

One relevant finding of the present study was the intracellular uptake and subcellular localization of EcTI in melanoma cells. EcTI was rapidly internalized and showed partial colocalization with intracellular compartments, including lysosomes and mitochondria, suggesting that its biological effects are not restricted to extracellular protease inhibition. Mitochondria are key regulators of cellular metabolism, oxidative stress responses, and regulated cell death pathways [12]. The simultaneous occurrence of mitochondrial depolarization, intracellular calcium dysregulation, and ROS accumulation indicates that EcTI profoundly impairs mitochondrial homeostasis rather than inducing isolated metabolic alterations. Because these processes are tightly interconnected, our findings further suggest that mitochondrial dysfunction may represent an important link between EcTI treatment and the activation of downstream stress-response and regulated cell death pathways.

Although moderate ROS production can promote tumor adaptation and survival, persistent oxidative stress leads to irreversible cellular damage and activates regulated cell death pathways [32]. Melanoma cells typically maintain elevated basal ROS levels while relying on robust antioxidant defenses to preserve viability. Under these conditions, additional ROS accumulation may exceed the adaptive threshold required for survival, converting oxidative signaling from a pro-tumorigenic mechanism into a trigger of irreversible cellular damage and regulated cell death. Moreover, mitochondrial dysfunction and Ca^2+^ imbalance are closely associated with activation of stress-response pathways and disruption of bioenergetic homeostasis, reinforcing the notion that metabolic collapse represents a central component of the antitumor activity of EcTI.

Autophagy is highly context-dependent in cancer biology and may either support tumor adaptation or contribute to cell death depending on stress intensity and cellular context [33]. In the present study, EcTI induced accumulation of acidic vesicular organelles together with marked alterations in autophagy-related proteins, including increased LC3-II/LC3-I ratio and modulation of ULK1, Ambra1, and Beclin-1 signaling. However, because autophagic flux was not directly evaluated, the observed accumulation of LC3-II cannot be interpreted exclusively as activation of productive autophagy. Instead, these findings may also reflect dysregulation of autophagic degradation and lysosomal processing, resulting in increased cellular stress. Similar interpretations have been proposed in studies demonstrating that excessive accumulation of autophagic markers may indicate defective autophagic flux rather than effective cytoprotective autophagy [34]. Nevertheless, these findings collectively suggest that EcTI interferes with proteostasis and intracellular stress adaptation pathways.

The induction of regulated cell death was confirmed by Annexin V/propidium iodide staining, caspase activation, mitochondrial dysfunction, and ultrastructural alterations. Partial rescue of viability after caspase inhibition supports the participation of apoptotic pathways, which are tightly associated with mitochondrial outer membrane permeabilization and stress signaling [35,36]. However, the EcTI-induced cytotoxicity does not appear to be fully explained by apoptosis alone.

Importantly, inhibition of necroptosis produced a more pronounced recovery of viability than caspase inhibition, suggesting that non-apoptotic death mechanisms may represent a major component of the EcTI response. This observation is particularly relevant because resistance to apoptosis is a hallmark of advanced melanoma, and activation of alternative regulated cell death pathways has emerged as an attractive therapeutic strategy for overcoming treatment resistance. Necroptosis is a regulated inflammatory form of cell death that is mainly driven by the RIPK1-RIPK3-MLKL axis and is often induced under conditions of metabolic stress, mitochondrial dysfunction, ROS accumulation, or impaired apoptotic signaling [37,38]. While further molecular validation is required to confirm activation of the canonical RIPK1-RIPK3-MLKL pathway, the functional evidence presented here is consistent with the involvement of necroptosis-like mechanisms in EcTI-induced melanoma cell death. This is particularly relevant as necroptosis has been identified as a potential therapeutic target in apoptosis-resistant tumors, such as melanoma [39].

Transmission electron microscopy further confirmed the coexistence of multiple modes of regulated cell death. Mitochondrial swelling, disorganization of cristae, vacuolization, chromatin condensation, and membrane blebbing are indicative of extensive cellular damage and severe metabolic stress. These ultrastructural observations are consistent with the notion that pathways of regulated cell death often overlap and interact rather than occurring as isolated processes [15,16]. Thus, EcTI appears to promote a multifactorial disruption of melanoma cell homeostasis through simultaneous perturbation of adhesion signaling, mitochondrial function, oxidative balance, and cell death regulation.

The ability of EcTI to suppress tumor growth without detectable systemic toxicity substantially increases its translational relevance. Although body weight alone does not provide a comprehensive toxicological assessment, EcTI maintained significant antitumor efficacy without detectable weight loss, suggesting a favorable safety profile under the experimental conditions used. The concentrations of EcTI used in vitro (5–100 μM) were selected based on preliminary dose–response experiments to characterize concentration-dependent biological effects under controlled conditions rather than to reproduce systemic exposure achieved in vivo. Likewise, the dosing regimen adopted in the murine melanoma model (4 and 8 mg/kg) was based on previous studies demonstrating antitumor efficacy with no evidence of systemic toxicity. Because pharmacokinetic and biodistribution studies were beyond the scope of the present work, a direct correlation between the effective in vitro concentrations and in vivo exposure cannot currently be established. Future pharmacokinetic and pharmacodynamic studies will be important to define tissue distribution, bioavailability, and the exposure–response relationship of EcTI, thereby supporting its translational development.

In addition, modulation of cytokines and chemokines involved in inflammation and immune regulation suggests that EcTI may also influence the tumor microenvironment beyond its direct effects on melanoma cells. Chronic inflammation and cytokine signaling are major contributors to melanoma progression and therapeutic resistance [40,41]. The reduction in pro-inflammatory mediators and modulation of chemokines involved in immune cell recruitment further support a potential immunomodulatory role of EcTI in the melanoma microenvironment. Although the in vitro findings provide plausible mechanistic explanations for the observed antitumor activity, the molecular pathways identified in cultured melanoma cells were not directly evaluated in tumor tissues. Therefore, future studies including immunohistochemical or biochemical analyses of tumor samples will be important to establish the in vivo contribution of these signaling pathways.

Several plant-derived Kunitz-type protease inhibitors have been investigated as promising anticancer molecules due to their ability to interfere with protease-dependent signaling, extracellular matrix remodeling, angiogenesis, and tumor cell invasion. Examples include BbKI from *Bauhinia bauhinioides*, soybean Kunitz trypsin inhibitor (SKTI), and other members of the Kunitz family that have demonstrated antiproliferative, anti-invasive, and anti-inflammatory activities in different experimental cancer models [19]. Consistent with these observations, EcTI also modulates multiple processes associated with tumor progression. While previous studies have largely focused on the characterization of cellular responses in vitro [21,22,23,24], the present work extends these findings by demonstrating that EcTI suppresses melanoma progression in a murine melanoma model in vivo. Together, these results further support the therapeutic potential of plant-derived Kunitz-type protease inhibitors as multifunctional candidates for cancer therapy.

The present study has some limitations that should be acknowledged. Although our data consistently demonstrate that EcTI modulates multiple pathways associated with melanoma progression, its direct molecular targets remain to be identified. Likewise, autophagic flux was not directly assessed, and activation of canonical necroptotic signaling was inferred from pharmacological inhibition rather than from direct evaluation of RIPK1, RIPK3, or MLKL phosphorylation. In addition, pharmacokinetic characterization, biodistribution, and molecular analyses of tumor tissues were beyond the scope of the present work, precluding direct correlation between the molecular alterations observed in vitro and those occurring within the tumor microenvironment in vivo.

Another limitation is that the mechanistic studies were performed using a single murine melanoma cell line (B16F10-Nex2). Although this model is widely used because of its aggressive phenotype and suitability for syngeneic in vivo studies, melanoma is a highly heterogeneous disease, and responses to EcTI may vary among different molecular subtypes. Human melanoma cell lines were not included in the present study because the antitumor effects of EcTI have already been demonstrated by our group in human melanoma cells, including inhibition of proliferation, adhesion, migration, invasion, and Src/FAK-dependent signaling pathways [24]. The objective of the present work was to extend these previous findings by investigating the antitumor activity of EcTI in a syngeneic murine melanoma model, enabling integration of mechanistic analyses with in vivo validation. In addition, the animal experiments were performed exclusively in male C57BL/6 mice to maintain consistency with previous studies using this well-established syngeneic melanoma model. Nevertheless, sex-related differences in tumor biology, immune responses, and therapeutic efficacy cannot be excluded and should be addressed in future studies.

Despite these limitations, the present work provides an integrated demonstration that EcTI suppresses melanoma progression in vivo while simultaneously affecting adhesion-associated signaling, mitochondrial homeostasis, oxidative stress, autophagy-associated pathways, regulated cell death, and systemic inflammatory mediators. Future studies aimed at identifying the direct molecular targets of EcTI, evaluating its pharmacokinetic profile and biodistribution, validating the proposed mechanisms in tumor tissues, and extending these findings to additional murine, human, and patient-derived melanoma models, including both sexes, will further strengthen the mechanistic understanding and translational potential of EcTI as a multitarget anticancer candidate.

## 4. Materials and Methods

### 4.1. Reagents

Dulbecco’s Modified Eagle Medium (DMEM), RPMI-1640 medium, fetal bovine serum (FBS), penicillin, streptomycin, trypsin-EDTA, Fluo-4 AM, Annexin V-FITC, and CellEvent™ Caspase-3/7 Green Detection Reagent were purchased from Thermo Fisher Scientific (Waltham, MA, USA). Fibronectin, laminin, vitronectin, collagen I, collagen IV, crystal violet, paraformaldehyde, Triton X-100, dimethyl sulfoxide (DMSO), acridine orange, tetramethylrhodamine ethyl ester (TMRE), propidium iodide (PI), and 2′,7′-dichlorofluorescin diacetate (DCFDA) were obtained from Sigma-Aldrich (St. Louis, MO, USA). Primary antibodies against FAK, phospho-FAK, Src, phospho-Src, ERK, phospho-ERK, Beclin-1, LC3-I/II, ULK1, and AMBRA1 were obtained from Cell Signaling Technology (Danvers, MA, USA) and Abcam (Cambridge, UK). HRP-conjugated secondary antibodies were obtained from Cell Signaling Technology (Danvers, MA, USA). All other reagents were of analytical grade.

### 4.2. Plant Material, EcTI Purification, and Experimental Design

Seeds of *Enterolobium contortisiliquum* were commercially obtained in São Paulo, Brazil. Since the material was acquired through commercial suppliers rather than collected from natural populations, no collection permits were required under Brazilian regulations. Species identification had been previously confirmed by a specialist at the Instituto Agronômico de Pernambuco (Recife, Brazil), where a voucher specimen (No. 61,415) is deposited.

EcTI was purified from *Enterolobium contortisiliquum* seeds as previously described [21], with minor modifications. Briefly, seed proteins were extracted by the salting-in method using 0.15 M NaCl at a 1:20 (*w*/*v*) ratio, followed by homogenization. The crude extract was heated at 50 °C for 10 min and centrifuged at 2254× *g* for 20 min at 4 °C. The resulting supernatant (saline extract) was subjected to precipitation with cold acetone (80%, *v*/*v*) under continuous stirring at 4 °C. After decantation for 30 min, the precipitated proteins were recovered by centrifugation (2254× *g* for 20 min at 4 °C), and residual acetone was allowed to evaporate at room temperature. The protein pellet was resuspended in 50 mM Tris-HCl buffer (pH 8.0), centrifuged under the same conditions, and the resulting supernatant was used for chromatographic purification.

The pH and conductivity of the sample were adjusted to match those of the equilibration buffer prior to chromatography. The protein extract was applied to a DEAE-Sepharose anion-exchange column (Cytiva, Piscataway, NJ, USA) previously equilibrated with 0.1 M Tris-HCl buffer (pH 8.0), using a loading capacity of 20 mg of protein per mL of resin. Unbound proteins were removed with the equilibration buffer until the absorbance at 280 nm returned to baseline. Bound proteins were sequentially eluted with the equilibration buffer supplemented with 0.03, 0.15, and 0.30 M NaCl. Chromatographic separations were performed at a flow rate of 1.0–1.5 mL/min, and the fractions containing trypsin-inhibitory activity were pooled.

The pooled fractions were further purified by affinity chromatography on a trypsin-Sepharose column previously equilibrated with 0.1 M Tris-HCl buffer (pH 8.0). After sample loading, the column was washed with equilibration buffer followed by 0.3 M NaCl until the absorbance at 280 nm returned to baseline. EcTI was subsequently eluted with 0.5 M KCl (pH 2.0), and the collected fractions were immediately neutralized with 1 M Tris-HCl (pH 9.0). Chromatography was performed at a flow rate of 0.7 mL/min. Fractions exhibiting absorbance at 280 nm were pooled, dialyzed against Milli-Q water, and lyophilized.

The EcTI-containing fractions obtained after trypsin-Sepharose chromatography were further purified by size-exclusion chromatography on a Superdex 75 10/300 GL column coupled to an ÄKTA Avant Fast Protein Liquid Chromatography (FPLC) system (Cytiva, NJ, USA). Prior to injection, the sample was filtered through a 0.45 μm membrane and centrifuged at 9000× *g* for 10 min. Elution was performed with 0.1 M Tris-HCl buffer (pH 8.0) containing 0.15 M NaCl at a constant flow rate of 0.3 mL/min. Protein-containing fractions were monitored by absorbance at 280 nm, pooled according to their chromatographic profile and trypsin-inhibitory activity, dialyzed against Milli-Q water, aliquoted, and stored at −20 °C until use.

The purity of EcTI was routinely monitored throughout the purification procedure by SDS-PAGE under non-reducing conditions (Supplementary Materials). The purified inhibitor migrated as a single protein band with an apparent molecular mass of approximately 20 kDa, consistent with previous biochemical characterization of EcTI [21,22]. Protein concentration was determined using the Bradford assay with bovine serum albumin as the standard.

To ensure experimental reproducibility, all biological assays were performed using EcTI obtained from the same purification batch. The purification protocol routinely yielded highly purified EcTI (>95% purity), as previously demonstrated by chromatographic profiles and electrophoretic analyses [24]. The biochemical identity and structural characterization of EcTI, including reverse-phase HPLC, MALDI-TOF mass spectrometry, SDS-PAGE, and its inhibitory profile against trypsin, plasma kallikrein, plasmin, neutrophil elastase, and factor XIIa, have been extensively reported in previous studies from our group [21,22,24]. Therefore, these analyses were not repeated for the present study. Before each experiment, aliquots were thawed only once and diluted in the appropriate experimental buffer to avoid repeated freeze–thaw cycles.

Preliminary dose–response assays previously performed by our group were used to establish the concentration range for in vitro experiments. Based on these analyses, EcTI concentrations from 5 to 100 μM were selected to evaluate dose-dependent effects on the viability, proliferation, adhesion, migration, invasion, mitochondrial function, intracellular signaling, autophagy and regulated cell death avoiding immediate nonspecific cytotoxicity, according to recommended practices for the screening of anticancer compounds [42].

### 4.3. In Vitro Functional Assays

#### 4.3.1. Cell Culture

B16F10-Nex2 murine melanoma cells, a non-commercial subline derived from B16F10 melanoma cells, were kindly provided by Prof. Dr. Jane Zveiter de Moraes (Department of Biophysics, Federal University of São Paulo-UNIFESP, São Paulo, Brazil). As this cell line is not commercially available, no accession number is available. Cells were cultured in RPMI-1640 medium supplemented with 10% heat-inactivated fetal bovine serum (FBS), 100 U/mL penicillin, 100 μg/mL streptomycin, 10 mM HEPES, and 24 mM sodium bicarbonate. Cells were maintained at 37 °C in a humidified atmosphere containisng 5% CO_2_ [43].

#### 4.3.2. Cell Viability and Proliferation Assays

Cell viability was evaluated by MTT reduction assay. B16F10-Nex2 cells were seeded in 96-well plates and allowed to adhere for 24 h before treatment with EcTI (5–100 μM) for 24, 48, or 72 h. MTT solution was then added and the resulting formazan crystals were solubilized in DMSO. Absorbance was measured spectrophotometrically and results were expressed as a percentage of untreated control cells [24,44].

Cell proliferation was assessed by BrdU incorporation assay. Cells (5 × 10^3^ cells/well) were treated with EcTI for 24, 48, or 72 h, and BrdU (10 μM) was added during the final 24 h of incubation. Following fixation and DNA denaturation, BrdU incorporation was detected using an anti-BrdU-peroxidase system (Roche, Basel, Switzerland). Chemiluminescence was measured using a FlexStation™ 3 Multi-Mode Reader (Molecular Devices, San Jose, CA, USA) [24].

#### 4.3.3. Cell Adhesion Assay

Cell adhesion assays were performed using extracellular matrix (ECM) proteins. Ninety-six-well plates were coated overnight at 4 °C with collagen I, collagen IV, laminin, fibronectin, or vitronectin at previously established concentrations. Wells were blocked with 1% bovine serum albumin (BSA) for 1 h at 37 °C.

B16F10-Nex2 cells (5 × 10^3^ cells/well) were preincubated with EcTI (5–100 μM) for 15 min and seeded onto ECM-coated wells. After 4 h incubation at 37 °C, non-adherent cells were removed by washing with PBS. Adherent cells were fixed with methanol, stained with toluidine blue, and solubilized with SDS. Absorbance was measured at 540 nm [45,46].

#### 4.3.4. Migration and Invasion Assays

Cell migration was assessed using a wound healing assay. Confluent monolayers were pretreated with mitomycin C to inhibit proliferation prior to scratch formation. A linear wound was generated using a sterile pipette tip, and cells were treated with EcTI (25–100 μM). Wound closure was monitored at 0 and 24 h and quantified using ImageJ software (version 1.53q; National Institutes of Health, Bethesda, MD, USA) [47,48].

Cell invasion assays were performed using Matrigel-coated Transwell chambers with 8 μm pore membranes. EcTI-treated cells suspended in serum-free medium were added to the upper chamber, while complete medium containing FBS was placed in the lower chamber as chemoattractant. After 24 h of incubation, invading cells were fixed, stained and counted microscopically [49].

#### 4.3.5. Gelatin Zymography and Assays Related to Angiogenesis

The activity of matrix metalloproteinase was determined by gelatin zymography. Conditioned media from EcTI (100 μM, 24 h) treated cells were collected and equal amounts of protein were run on SDS-PAGE gels containing gelatin. Gels were stained with Coomassie Brilliant Blue after renaturation and incubation in activation buffer and proteolytic activity was visualized as clear bands on the stained background [50].

The angiogenic potential of conditioned media derived from EcTI-treated melanoma cells was evaluated using an endothelial tube formation assay. Human umbilical vein endothelial cells (HUVECs) were seeded onto Matrigel-coated plates and incubated with conditioned media from control or EcTI-treated B16F10-Nex2 cells. After 24 h, capillary-like structures were analyzed by phase-contrast microscopy [51].

#### 4.3.6. Confocal Microscopy and High-Content Imaging

EcTI internalization was investigated using Alexa Fluor^TM^ 488-conjugated EcTI, prepared according to the manufacturer’s instructions (Thermo Fisher Scientific, Waltham, MA, USA). Cells were incubated with labeled EcTI (100 μM) and counterstained with organelle-specific fluorescent probes. Images were acquired using a Leica TCS SP8 confocal microscope (Leica Microsystems, Wetzlar, Germany). Images were acquired using the ImageXpress^®^ Micro Confocal High-Content Imaging System (Molecular Devices, San Jose, CA, USA). A total of 16 fields per well were acquired at 20× magnification. Image Analysis was subsequently performed using the MetaXpress^®^ Acquisition & Analysis Software version 6.7.2.290 (Molecular Devices, San Jose, CA, USA). Data obtained by HCS represent fluorescence intensity relative to secondary-only controls.

#### 4.3.7. Western Blot Analysis

Cells treated with EcTI (25–100 μM) were lysed in RIPA buffer supplemented with protease and phosphatase inhibitors. Protein concentrations were determined using the Bradford assay, and equal amounts of protein were separated by SDS-PAGE and transferred onto PVDF membranes. Membranes were incubated overnight at 4 °C with primary antibodies against FAK, phospho-FAK, Src, phospho-Src, ERK, phospho-ERK, Bcl-2, Bax, Beclin-1, LC3-I/II, ULK1, and AMBRA1. Following incubation with HRP-conjugated secondary antibodies, immunoreactive bands were visualized by enhanced chemiluminescence. Densitometric analyses were performed using ImageJ software (version 1.53q; National Institutes of Health, Bethesda, MD, USA, normalized to GAPDH, and expressed relative to untreated controls [52].

#### 4.3.8. Detection of Acidic Vesicular Organelles

Autophagic vesicles were detected by acridine orange staining [34,53]. Cells were treated with EcTI (50 or 100 μM) for 24 h, while EBSS starvation medium was used as positive control. Cells were stained with acridine orange (0.5 μg/mL) for 15 min and analyzed using a Leica DMi8 fluorescence microscope. Acidic vesicular organelles were evaluated by red fluorescence emission.

#### 4.3.9. Mitochondrial Membrane Potential (ΔΨm)

Mitochondrial membrane potential was assessed using TMRE, a membrane potential-sensitive fluorescent dye [54]. B16F10-Nex2 cells were treated with EcTI (50 and 100 μM) for 24 h and subsequently incubated with TMRE (100 nM) for 45 min at 37 °C in the dark. FCCP (50 μM) was used as positive control for mitochondrial depolarization.

Fluorescence images were acquired using a Leica DMi8 fluorescence microscope (Leica Microsystems, Wetzlar, Germany), and fluorescence intensity was quantified using ImageJ software (version 1.54p; National Institutes of Health, Bethesda, MD, USA) as corrected total cell fluorescence (CTCF).

#### 4.3.10. Intracellular Ca^2+^ Measurement

Intracellular calcium levels were measured using Fluo-4 AM fluorescent probe according to previously established protocols [55]. Cells were treated with EcTI (25–100 μM) for 24 h, washed with PBS, and incubated with Fluo-4 Direct Calcium reagent containing probenecid. Fluorescence was measured using a FlexStation™ 3 Multi-Mode Reader (Molecular Devices) and analyzed as relative fluorescence units (RFU).

#### 4.3.11. Intracellular Reactive Oxygen Species (ROS) Production

Intracellular ROS production was assessed using CM-H2DCFDA fluorescent probe. Cells were treated with EcTI (50 or 100 μM) for 24 h, while H_2_O_2_ (500 μM) was used as positive control. Following incubation with CM-H2DCFDA (5 μM), fluorescence was analyzed by flow cytometry using a BD Accuri C6 cytometer (BD Biosciences, San Jose, CA, USA).

Complementary fluorescence quantification was performed in black 96-well plates using DCFDA (10 μM), and fluorescence intensity was measured with a FlexStation™ 3 reader at excitation/emission wavelengths of 485/530 nm [56].

#### 4.3.12. Cell Death Analysis

Cell death was evaluated by Annexin V-FITC/PI staining followed by flow cytometry. Cells treated with EcTI (5–100 μM) for 24 h were detached using Versene solution, washed with PBS, and incubated with Annexin V-FITC and PI in binding buffer for 15 min at room temperature in the dark. At least 10,000 events were acquired using a BD Accuri C6 cytometer. Staurosporine (1 μM) and Triton X-100 (0.1%) were used as positive controls for apoptosis and necrosis, respectively [57].

Nuclear morphology was analyzed by Hoechst 33342 staining. Cells treated with EcTI (50 and 100 μM) were incubated with Hoechst 33342 (0.1 μg/mL) for 10 min and visualized using fluorescence microscopy [58].

Loss of plasma membrane integrity was assessed by propidium iodide uptake assay. Cells were treated with EcTI (25–100 μM) or staurosporine (1 μM), stained with PI (5 μg/mL), and analyzed by fluorescence microscopy [58].

Caspase-3/7 activation was analyzed using CellEvent™ Caspase-3/7 Green Detection Reagent according to the manufacturer’s instructions.

#### 4.3.13. Transmission Electron Microscopy (TEM)

Ultrastructural analysis was performed by transmission electron microscopy as previously described [24]. Cells treated with EcTI (100 μM, 24 h) were fixed with 2% formaldehyde and 2.5% glutaraldehyde in sodium cacodylate buffer, post-fixed with osmium tetroxide, dehydrated in graded ethanol, and embedded in Epon resin.

Ultrathin sections (70 nm) were obtained using an ultramicrotome, stained with uranyl acetate and lead citrate, and examined using a JEOL JEM-1200 EX II transmission electron microscope (Tokyo, Japan) operated at 80 kV. Images were recorded with a Gatan 791 CCD camera (Pleasanton, CA, USA).

### 4.4. In Vivo Studies

#### 4.4.1. Animal Model

Male C57BL/6 mice (7–8 weeks old) were subcutaneously inoculated with 1 × 10^5^ B16F10-Nex2 melanoma cells. Animals were randomly divided into experimental groups and treated daily with intraperitoneal injections of EcTI (4 or 8 mg/kg) or vehicle control. Tumor volume and body weight were monitored every two days. Animals were euthanized when tumors reached approximately 1500 mm^3^.

All experimental procedures were approved by the Ethics Committee of the Universidade Federal de São Paulo (protocol #7812290720) and conducted according to institutional guidelines for animal experimentation.

#### 4.4.2. Cytokine Analysis

Plasma cytokine and chemokine levels were determined using the MAGPIX Multiplexing System (Luminex Corporation, Austin, TX, USA). Plasma samples were incubated with fluorescent bead-conjugated antibodies specific for each cytokine, followed by incubation with biotinylated secondary antibodies and streptavidin-phycoerythrin. Data acquisition and analysis were performed using xPONENT 4.2 software (Luminex Corporation, Austin, TX, USA), and cytokine concentrations were determined from standard curves [59].

### 4.5. Statistical Analysis

All experiments were independently performed at least three times. Data are presented as mean ± SD or SEM, as indicated in the corresponding figure legends. Statistical analyses were performed using GraphPad Prism software 8.2.1 (GraphPad Software, Boston, MA, USA).

Data distribution was assessed for normality prior to statistical analysis. Comparisons among multiple groups were performed using one-way or two-way analysis of variance (ANOVA), followed by Tukey’s or Dunnett’s multiple comparisons tests, as appropriate. Differences were considered statistically significant at *p* < 0.05.

## 5. Conclusions

The present study demonstrates that the plant-derived Kunitz-type protease inhibitor EcTI exerts significant antitumor activity against melanoma by modulating multiple biological processes associated with tumor progression. EcTI reduced melanoma cell adhesion, migration, invasion, and proliferation, while suppressing FAK/Src/ERK signaling involved in adhesion-dependent cellular responses. These early effects were followed by mitochondrial dysfunction, intracellular calcium imbalance, oxidative stress, alterations in autophagy-related pathways, and activation of regulated cell death mechanisms, culminating in reduced tumor growth in vivo without detectable systemic toxicity.

**Figure 13:**
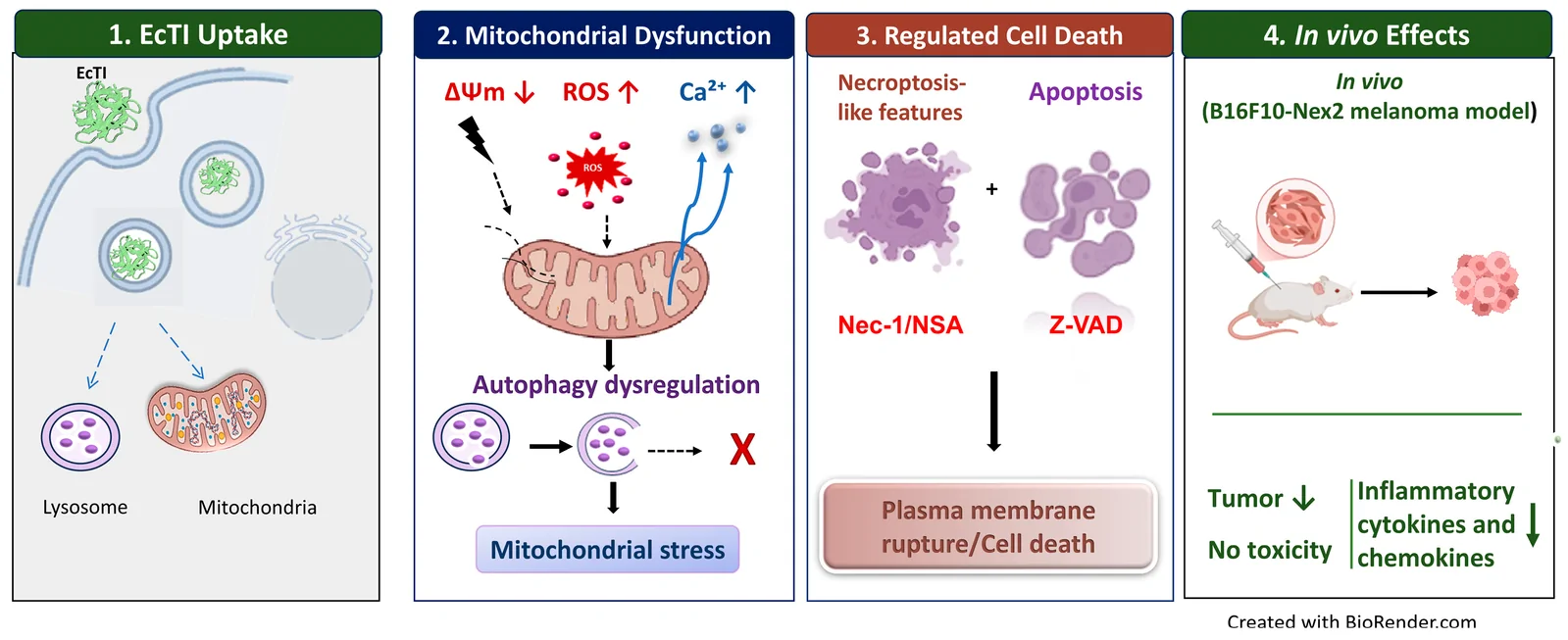
Schematic summary of the main findings of this study. EcTI is internalized by B16F10-Nex2 melanoma cells and associated with lysosomal and mitochondrial compartments. EcTI modulates focal adhesion and proteolytic pathways, autophagy-related processes, mitochondrial function, ROS production, and intracellular calcium, leading to apoptotic and necroptosis-like features and modulation of inflammatory mediators. These effects contribute to melanoma suppression in vitro and in vivo without detectable systemic toxicity. The model represents a working hypothesis, as the direct molecular target(s), quantitative colocalization, and autophagic flux remain to be determined.

Collectively, our findings indicate that EcTI modulates both intrinsic tumor cell signaling and tumor–microenvironment interactions, contributing to the inhibition of melanoma progression in vitro and in vivo. In addition to its direct effects on melanoma cells, EcTI modulated inflammatory cytokines and chemokines associated with the tumor microenvironment, further supporting its broad antitumor activity (Figure 13).

Although additional studies are required to identify the direct molecular targets of EcTI, characterize its pharmacokinetic properties, and further define the mechanisms regulating autophagy and necroptosis-like cell death, the present work provides important mechanistic insight into the antitumor activity of this plant-derived protease inhibitor. These findings support EcTI as a promising candidate for further preclinical development, either as a single agent or in combination with current therapeutic strategies for melanoma.

## Data Availability

All primary data from this paper are available from the corresponding author upon request.

## Supplementary Materials

The following supporting information can be downloaded at https://www.mdpi.com/article/10.3390/molecules31162829/s1.
