# Supplementary material for: The Plant Protease Inhibitor EcTI Suppresses Melanoma Progression In Vivo
Source: Molecules. 2026 Aug 13;31(16):2829. doi: 10.3390/molecules31162829 (PMC13515760; doi:10.3390/molecules31162829)
Supplement: Supplementary file 1 [file molecules-31-02829-s001.zip › molecules-4481028-supplementary.pdf]

The Plant Protease Inhibitor EcTI Suppresses Melanoma Progression *In Vivo*

Camila Ramalho Bonturi[1], Bruno Ramos Salu[1], Kathleen Chwen Ming Lie[1], Marcia Bonini[1], Rita de C. Sinigaglia[2], Miryam P. Alvarez-Flores[3], Ana M. Chudzinski-Tavassi[3], Heloisa Sobreiro Selistre-de-Araujo[4], Maria Luiza Vilela Oliva[a1*]

[1]Department of Biochemistry, Universidade Federal de São Paulo (UNIFESP), São Paulo 04044-020, Brazil

[2]Electron Microscopy Center, Universidade Federal de São Paulo (UNIFESP), São Paulo 04044-020, SP, Brazil

[3]Centre of Excellence in New Target Discovery (CENTD), Instituto Butantan, São Paulo 05503-900, SP, Brazil

[4]Department of Physiological Sciences, Universidade Federal de São Carlos, Rodovia Washington Luis, Km 235, São Carlos, SP, 13565-905, Brazil

*Correspondence:

Maria Luiza Vilela Oliva

mlvoliva@unifesp.br

Supplementary Figure S1. Biochemical characterization and purity assessment of the EcTI batch used in this study.

(A) Size-exclusion chromatography of purified EcTI on a Superdex 75 10/300 GL column. (B) Reverse-phase HPLC chromatogram of the purified EcTI preparation on a C18 column. (C) SDS-PAGE under non-reducing conditions showing a single protein band of approximately 20 kDa. Together, these analyses confirm the homogeneity and purity (>95%) of the EcTI batch used in all *in vitro* and *in vivo* experiments.

(A)

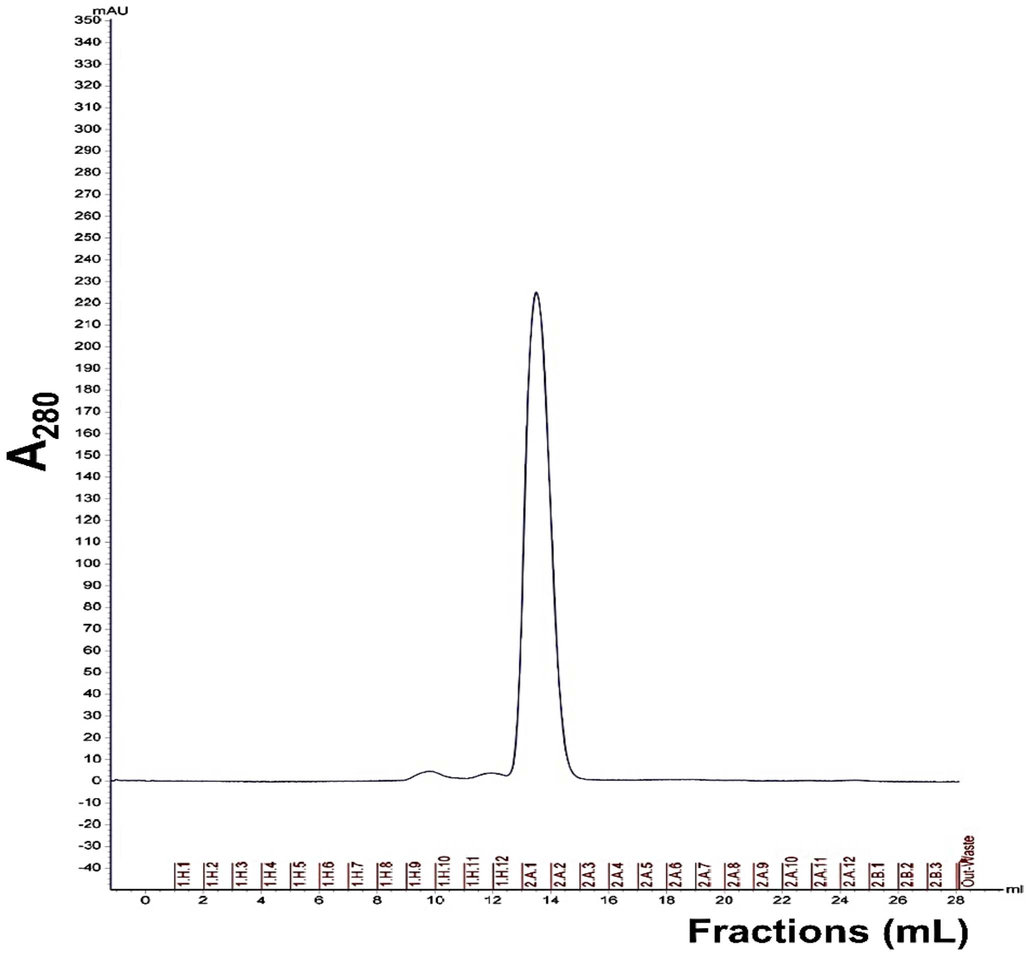

Figure S1 (A). Molecular Exclusion Chromatography of EcTI. Sample: EcTI from Trypsin-Sepharose eluted KCl/HCl 0.5 M, pH 2.0, with subsequent neutralization with 50 µL of 1 M Tris/HCl buffer, was applied into Superdex-75 10/300 GL. The column was equilibrated with Tris/HCl 0.05 M buffer, pH 8, plus NaCl 0.15 M coupled to an ÄKTA Avant 25 (Cytiva). The EcTI fraction was pooled, dialyzed, lyophilized, and applied on a High-Performance Liquid Chromatography into a C18 column. The solid black line is the 280 nm reading. Fractions of 1 mL were collected under a constant flow rate of 0.5 mL/min.

(B)

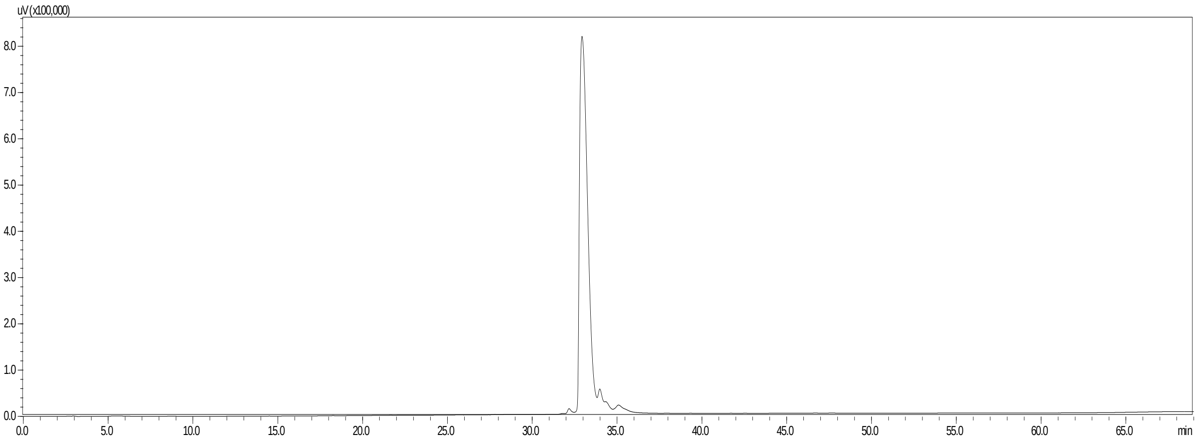

Figure S1 (B). High-Performance Liquid Chromatography of EcTI. The fraction obtained by molecular exclusion chromatography was submitted to reverse phase chromatography on a C18 HPLC column (15 cm × 4.6 mm; Vydac). The protein fraction was eluted with a linear gradient (5–100%) of 90% (v/v) acetonitrile in 0.1% (v/v) TFA in milli-Q water at the flow rate of 0.7 mL/min (t=0.1 min, 5% B; t=5 min, 5% B; t=30 min, 40% B; t=50 min, 50% B; t=60 min, 100% B; t=65–68 min, 0% B).

(C)

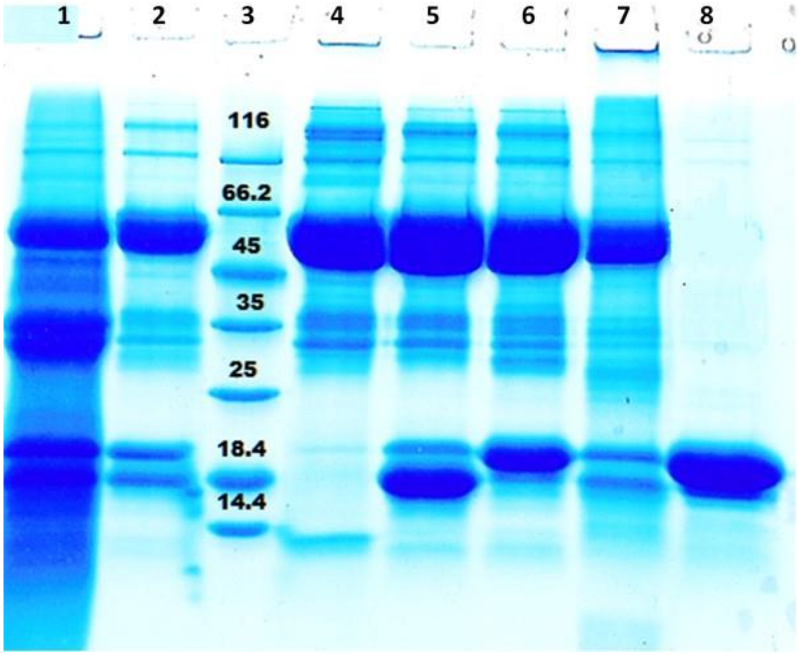

Figure S1 (C). Purification of EcTI from *Enterolobium contortisiliquum* seeds monitored by SDS-PAGE under non-reducing conditions. Lane **1**, crude saline extract; lane **2**, 80% acetone precipitate; lane **3**, molecular mass markers (kDa); lane **4**, unbound fraction from the DEAE-Sephadex column; lane **5**, proteins eluted with 0.035 M NaCl in 0.1 M Tris-HCl buffer (pH 8.0); lane **6**, proteins eluted with 0.15 M NaCl; lane **7**, proteins eluted with 0.30 M NaCl; lane **8**, purified EcTI after size-exclusion chromatography (Superdex 75 10/300 GL). The electrophoretic profile demonstrates the progressive enrichment of EcTI throughout the purification procedure and the high purity of the final preparation used in all *in vitro* and *in vivo* experiments.

Supplementary Figure S2. Original uncropped Western blot membranes used for quantitative analyses.

Representative examples of the original membranes used for protein quantification in the manuscript. The positions of membrane sectioning and the proteins analyzed in each membrane segment are indicated.

For the Western blot analyses, membranes were initially transferred and processed as intact blots. After transfer, each membrane was cut into sections according to the molecular weight ranges of the proteins of interest, allowing the detection of multiple targets on the same original blot. All membrane sections originated from the same membrane and were processed under identical experimental conditions. Unless otherwise indicated, all sections were developed simultaneously using the same exposure settings, ensuring consistency and comparability among the analyzed proteins.

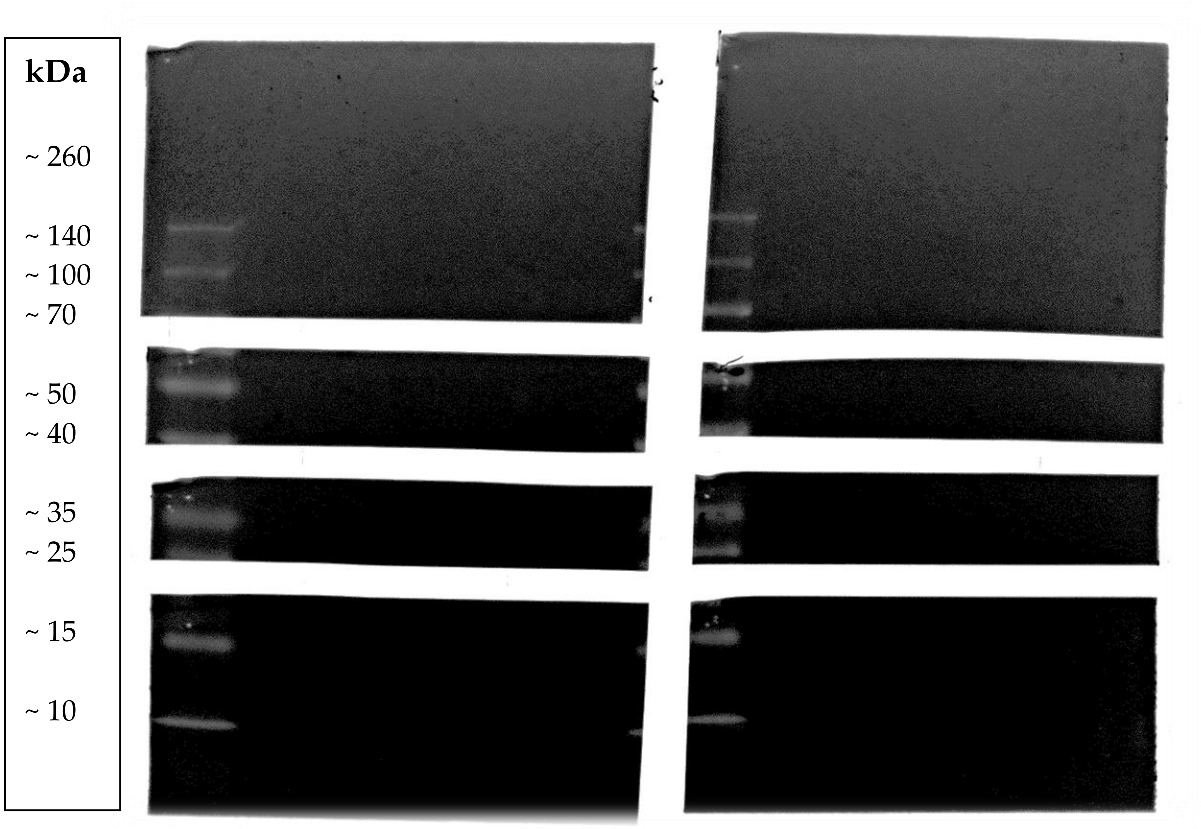

All protein bands presented in the manuscript originated from the same original membrane. Any apparent discontinuities reflect membrane sectioning according to molecular weight ranges after transfer and do not represent separate experiments or image manipulation.

Only the right portion of the membrane containing GAPDH was used for analysis, as illustrated below.

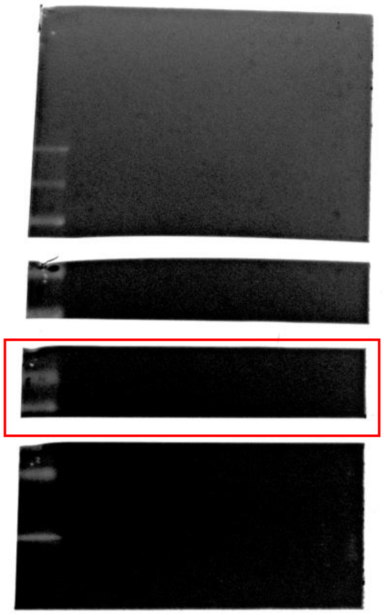

The entire gel with the cuts membranes – to see the ladder

AMBRA

P-AKT

GAPDH

CITOCROMO C

Map of the gel, considering the specific protein to each cut membrane.

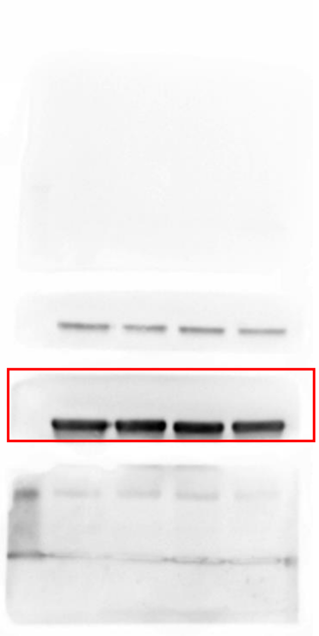

37 kDa

The GAPDH was used as representative for the western blotting.

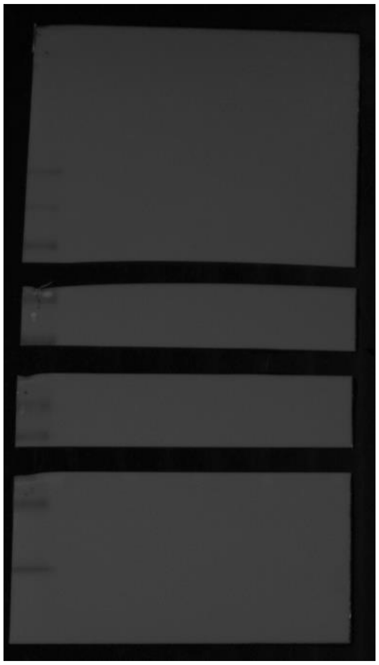

GAPDH was used as the loading control and served as the reference for normalization of all proteins analyzed from the same membrane.

Detection of AMBRA1 required a longer exposure time than that used for proteins such as p-PI3K, GAPDH, and BAX due to its lower signal intensity. The AMBRA1 blot shown here corresponds to the exposure used for quantitative analysis in the manuscript.

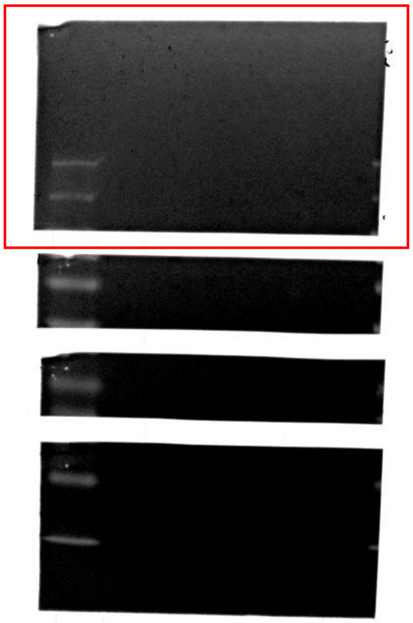

The entire gel with the cuts membranes – to see the ladder

AMBRA

P-PI3K

GAPDH

BAX

Map of the gel, considering the specific protein to each cut membrane.

140 kDa

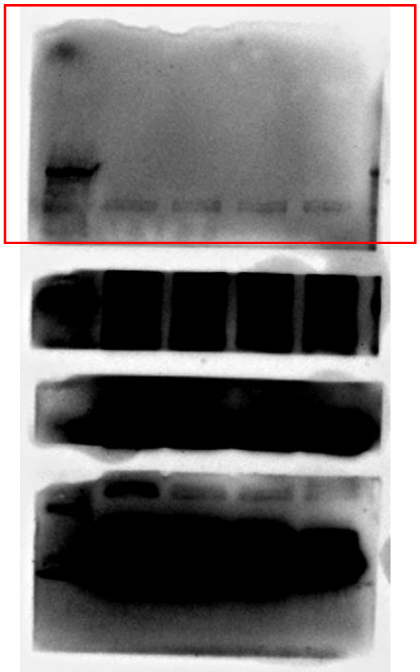

The **AMBRA** was used as representative for the western blotting.

Note: Not all proteins detected on the original membrane were included in the final manuscript

The same procedure was applied to Beclin-1, as shown below.

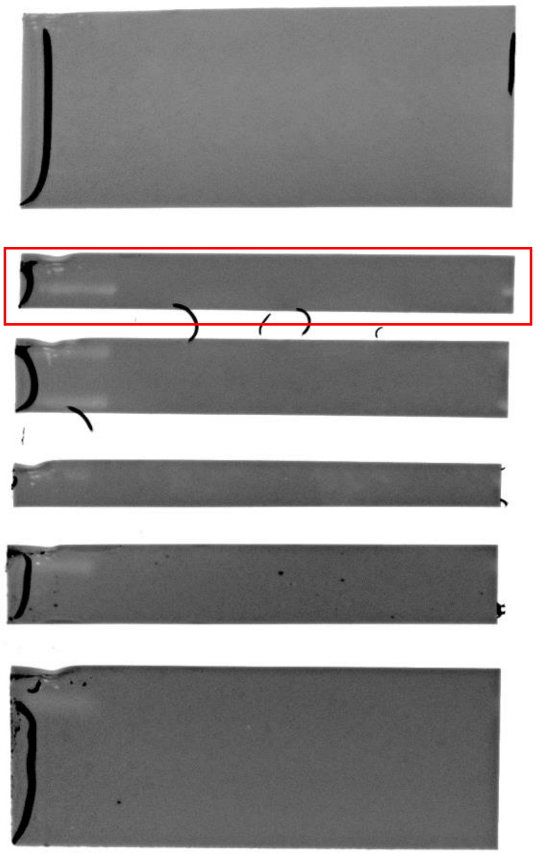

The entire gel with the cuts membranes

Ambra

p-FAK

Beclin

Par4

Bcl2

60 kDa

Map of the gel, considering the specific protein to each cut membrane.

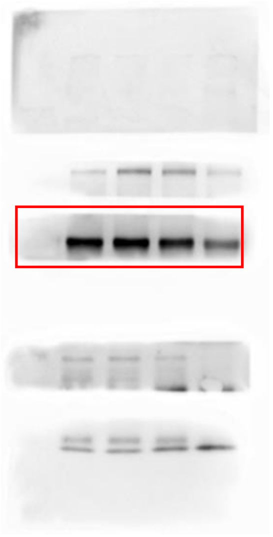

The **Beclin-1** blot shown here is representative of the membrane used for quantitative analysis in the manuscript.

The same approach was used for phospho-Beclin-1, as shown below.

The entire membrane is shown; however, only the left portion containing the molecular weight range corresponding to phospho-Beclin-1 was used for analysis.

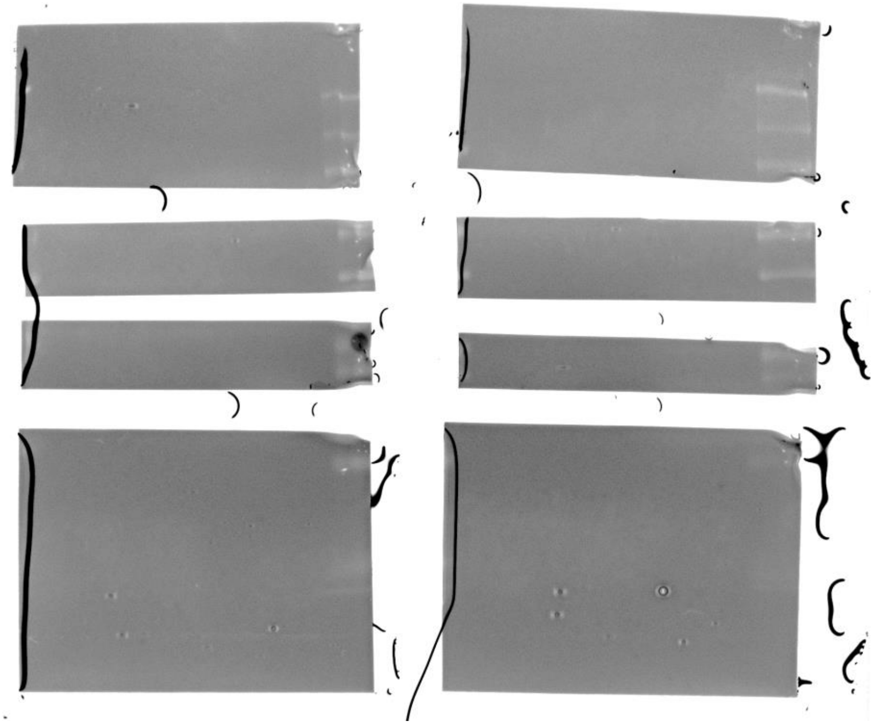

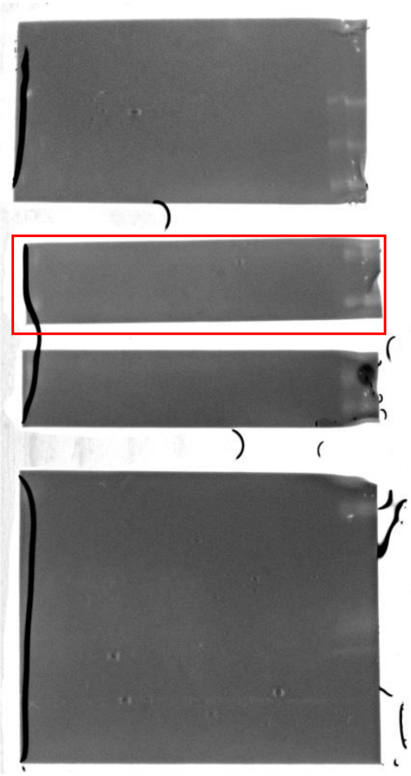

The entire gel with the cuts membranes

ULK

p-beclin

Par4

LC3

Map of the gel, considering the specific protein to each cut membrane.

60 kDa

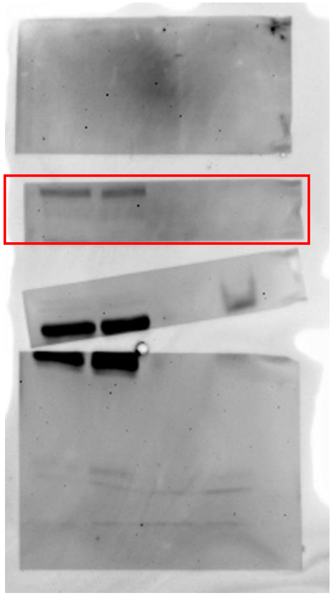

The **phospho-Beclin-1** blot shown here is representative of the membrane used for quantitative analysis in the manuscript.

The same with ULK:

The entire membrane is shown; however, only the left portion containing ULK1 was used for quantitative analysis.

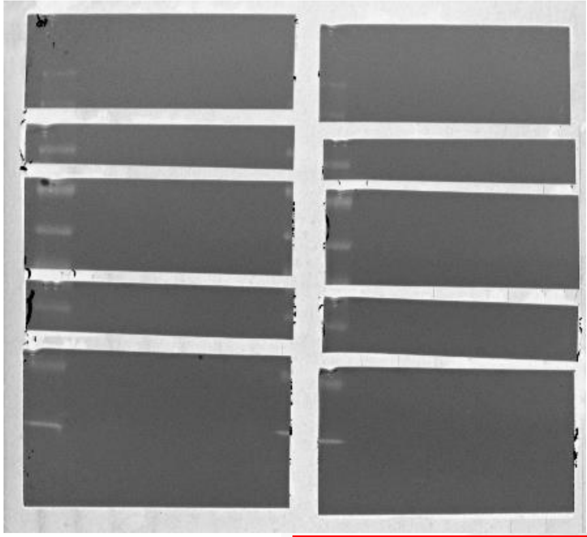

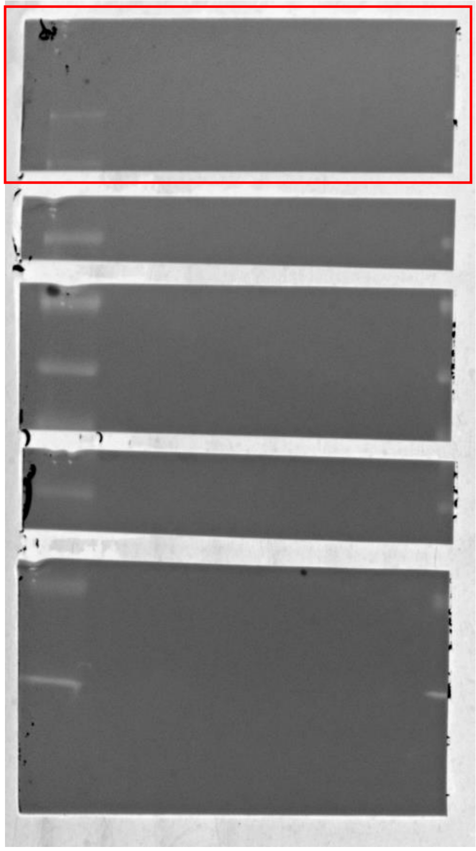

The entire gel with the cuts membranes

ULK **~140 kDa** →

FAK →

Beclin →

Par4 →

Cito *c* →

Map of the gel, considering the specific protein to each cut membrane.

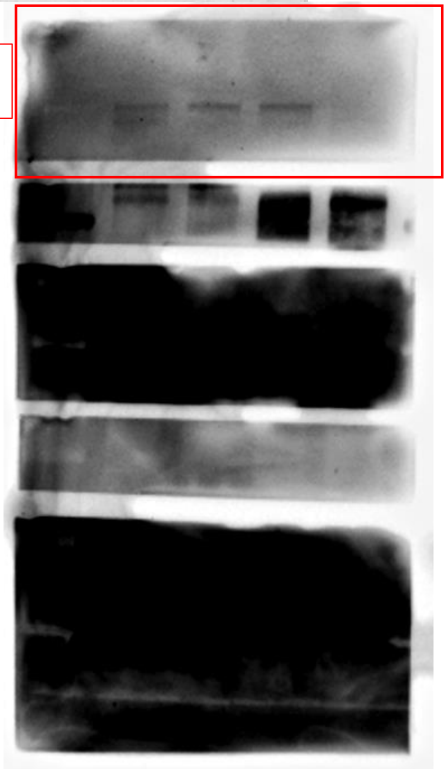

The **ULK1** blot shown here is representative of the membrane used for quantitative analysis in the manuscript.

The same with LC3:
The entire membrane is shown; however, only the left portion containing LC3-I and LC3-II was used for quantitative analysis.

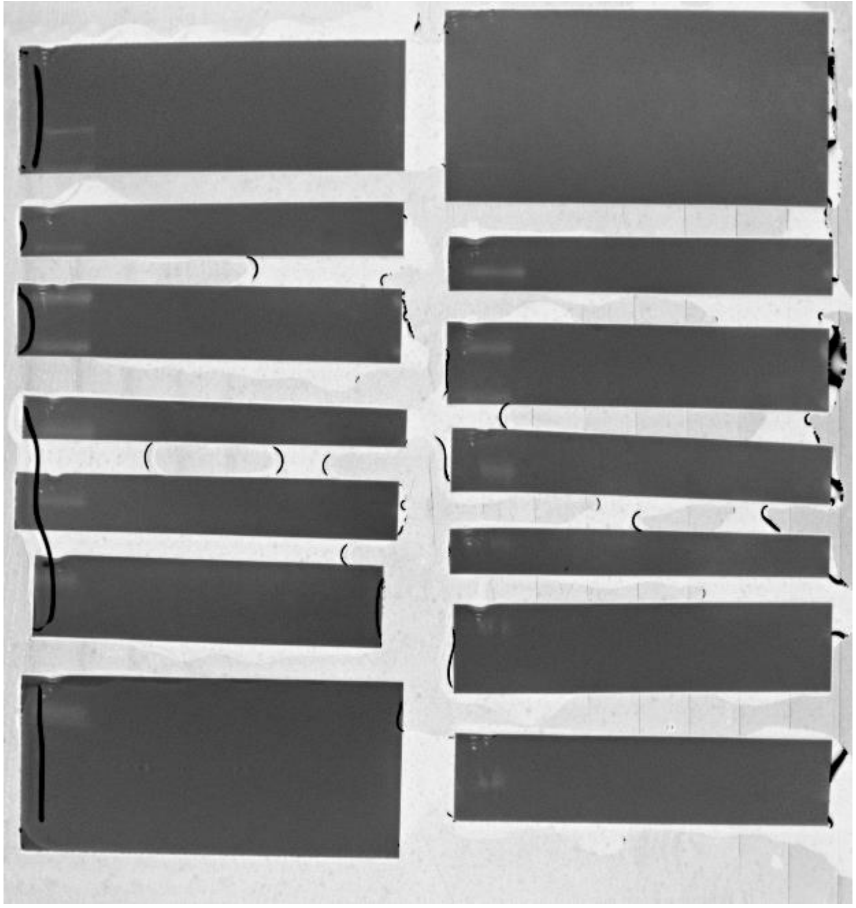

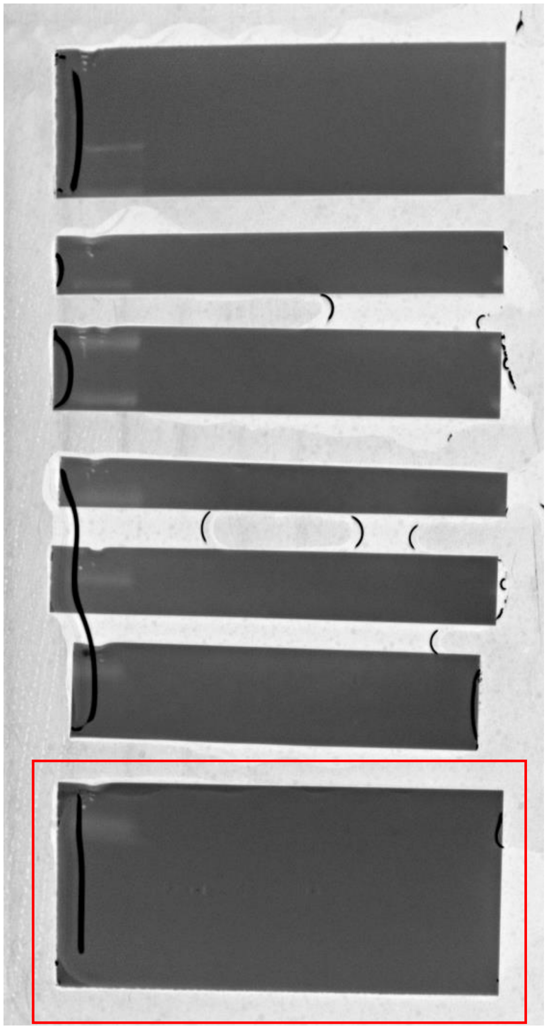

The entire gel with the cuts membranes

Map of the gel, considering the specific protein to each cut membrane.

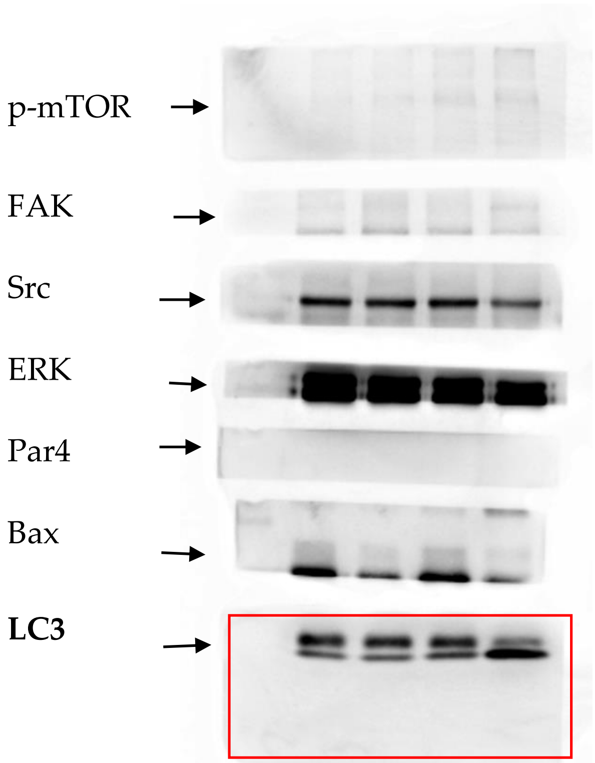

The **LC3** blot shown here is representative of the membrane used for quantitative analysis in the manuscript.

LC3-I ~ 16/18 kDa
LC3-II ~ 14/16 kDa
